# Solving the vibrational Schrödinger equation with artificial neural networks

**DOI:** 10.1038/s41467-026-74537-4

**Published:** 2026-06-20

**Authors:** Shuaishuai Zhao, Dong H. Zhang

**Affiliations:** 1 State Key Laboratory of Chemical Reaction Dynamics, Dalian Institute of Chemical Physics, Chinese Academy of Sciences, Dalian, China; 2 University of Chinese Academy of Sciences, Beijing, China; 3 Hefei National Laboratory, Hefei, China

**Keywords:** Method development, Chemical physics, Physical chemistry

## Abstract

Artificial neural networks are universal function approximators and have shown great ability in computing the ground-state energy of the electronic Schrödinger equation, yet have not established themselves as a practical and accurate approach for solving the vibrational Schrödinger equation for realistic polyatomic molecules. Here, we propose an efficient neural-network approach for solving the vibrational Schrödinger equation and provide a detailed illustration using the methane molecule. To demonstrate the power of the proposed method, we then apply it to propane, an 11-atom molecule with 27 vibrational degrees of freedom. Using a neural network with fewer than 15,000 parameters, we obtain the ground-state energy within 1 cm^−1^ of the reference value obtained from a diffusion Monte Carlo calculation, as well as vibrational energies for three excited states involving C-C-C stretching/bending modes that agree with the corresponding experimental values within the experimental uncertainties. The proposed method is expected to provide highly accurate vibrational energies and wavefunctions for molecules with more than 20 atoms.

## Abstract (web summary)

Solving the full-dimensional vibrational Schrödinger equation remains challenging for polyatomic molecules. The authors present an efficient neural-network approach for accurate vibrational energy calculations for molecules with more than ten atoms.

## Introduction

The importance of solving the time-independent Schrödinger equation (SE) for quantum systems needs no introduction. However, the equation can only be solved analytically for hydrogen-like atoms and some model problems. Tremendous efforts have been devoted to developing efficient methods to solve the equation numerically for increasingly large systems with the help of modern computer technology. The approach used most widely and successfully is to expand the wave function (WF) in terms of some basis functions, thereby converting the equation into a linear algebra problem. Unfortunately, the size of basis functions grows exponentially with the dimensionality of a system, leading to a crisis known as the curse of dimensionality.

Over the past decades, this rigorous quantum approach has been evolving substantially through the development of increasingly efficient basis functions and methods for evaluating kinetic energy operators^1–20^. It is now feasible to perform fully variational computations for various small molecules containing up to six atoms in full dimensions. Approaches based on the Smolyak sparse-grid scheme have been developed and successfully applied to full-dimensional vibrational calculations on malonaldehyde, a molecule with nine atoms^21–23^. Beyond these fully variational full-dimensional calculations, many approximate methods, most originally developed for electronic structure calculations, have been applied to compute vibrational energy levels of medium- to large-sized molecular systems with respectable accuracy^24–33^. These methods are typically formulated within a Watson-type Hamiltonian framework and employ truncated representations of the potential energy surface, such as n-mode or Taylor expansions around the equilibrium geometry, while incorporating rotation–vibration interactions through the kinetic-energy operator^27,29,30,32–34^.

Artificial neural networks (NNs) are universal function approximators and can be used to efficiently represent high-dimensional solutions for partial differential equations^35–39^ and WFs for many-body quantum systems with exponentially scaling complexity^40–43^. Great progress has been made in exploring NNs for the solution of the electronic SE^44–51^. Deep neural network electronic WF ansatzes, such as FermiNet^46^, PauliNet^47^, QianKunNet^49,50^, and SchrödingerNet^51^, have been proposed to achieve highly accurate solutions of the electronic SE.

In the past decades, neural networks have also been used to solve the vibrational SE^52–63^. Lagaris et al. studied a three-dimensional system of coupled anharmonic oscillators, which is comparable to the treatment of a non-linear three-atomic system^53^. Nakanishi and Sugawara considered a one-dimensional double-well potential linked to quantum tunneling in addition to the harmonic oscillator^54^. Mills and coworkers demonstrated their methods on systems including two-dimensional double-well inverted Gaussian potentials as well as entirely random two-dimensional potential grids^57^. Hong et al. applied their functional tensor network to a three-body coupled oscillator, thus giving rise to correlation and entanglement^60^. Gamper et al. applied a feed-forward neural network to one-dimensional anharmonic oscillators, rigid rotors, periodic finite potentials, as well as the hydrogen molecule and the torsional tunneling of the phenol molecule^61^. Domingo et al. considered one-dimensional anharmonic and polynomial potentials^62^. Manzhos et al. solved the vibrational SE for H_2_O by combining non-linear optimization of NN parameters with a linear matrix method^64^. Therefore, despite all these advances, NN has not established itself as a spectroscopic approach to solve the vibrational SE for realistic polyatomic molecules. One factor that makes calculations of vibrational eigenstates for realistic polyatomic molecules cumbersome is the evaluation of kinetic energy operator (KEO). Radau coordinates used in the case of the water molecule, are simple to apply the exact KEO analytically to NNs for a triatomic molecule^47^, but not for large molecules. The other problems mentioned above used simple KEOs based on second derivatives. In addition, in contrast to solving the electronic SE which mainly concerns the ground state energy, it is more challenging to solve the vibrational SE for the excited states because the vibrational ground state can be solved easily by the diffusion Monte Carlo method^65^.

The common approach to solving the SE with NNs is to represent a WF as an NN and optimize its parameters by minimizing the following loss function in NN training,

1 $$R=\frac{\left\langle \psi | {(\hat{H}-E)}^{2} | \psi \right\rangle }{\left\langle \psi | \psi \right\rangle }\approx \frac{{\sum }_{k=1}^{M}{\left|\left.\left(\hat{H}-E\right)\psi \left({\vec{{{{\boldsymbol{r}}}}}}_{k}\right)\right|\right.}^{2}}{{\sum }_{k=1}^{M}{\left|\left.\psi \left({\vec{{{{\boldsymbol{r}}}}}}_{k}\right)\right|\right.}^{2}}$$

where$\,\hat{H}$ is the Hamiltonian of a quantum system,$\,\psi$ is the WF represented by the NN, *E* is the expectation value of the Hamiltonian calculated as

2 $$E=\frac{\left\langle \psi | \hat{H} | \psi \right\rangle }{\left\langle \psi | \psi \right\rangle }\approx \frac{\mathop{\sum }_{k=1}^{M}\psi \left({\vec{{{{\boldsymbol{r}}}}}}_{k}\right)\hat{H}\psi \left({\vec{{{{\boldsymbol{r}}}}}}_{k}\right)}{\mathop{\sum }_{k=1}^{M}{\left|\left.\psi \left({\vec{{{{\boldsymbol{r}}}}}}_{k}\right)\right|\right.}^{2}}$$

where ${\vec{{{{\boldsymbol{r}}}}}}_{k}$ are the quadrature points or Monte Carlo (MC) sampling points to converge the configuration space integration, *M* is the corresponding number of points. The use of quadrature points in loss function evaluation actually does not circumvent the curse of dimensionality for the SE. On the other hand, the large number of MC sampling points required to converge the integration makes the NN parameter optimization expensive even using the stochastic gradient descent method to randomly select a small batch of MC points at each iteration^66^. To obtain eigenvalues and WFs for the excited states, one usually has to add an additional term in Eq. (1) to ensure orthogonality of the WFs^36,42^.

Here, we propose a strategy to solve the vibrational SE for polyatomic molecules by NNs using internuclear distances as the input coordinates for a molecular configuration. Molecular configurations for NN parameter optimization are selected not based on the requirements for WF integration as in Eq. (1), but according to the training error of the local energy. By initializing an NN with the normal mode WF for a target state of a molecule, we can obtain an accurate energy and WF for the state without imposing any orthogonality condition. Because the Hamiltonian operator for a molecular system is a Hermitian operator, the calculated WFs are mutual orthogonal for non-degenerate eigenstates within numerical uncertainty, provided they are sufficiently accurate. We demonstrate the proposed method on the methane (CH_4_) molecule, obtaining spectroscopically accurate energies for the ground and several excited states. To further demonstrate its power, we also apply it to the propane (C_3_H_8_) molecule. The calculated ground-state energy is within 1 cm⁻¹ of the reference diffusion Monte Carlo (DMC) value. Moreover, the vibrational energies for three C–C–C stretching/bending excited states agree with the corresponding experimental values within experimental uncertainties.

## Results and discussion

Now we use solving the first antisymmetric stretch excited state of the CH_4_ molecule on the potential energy surface constructed by Wang and Carrington^67^ as an example to illustrate the whole procedure. The feed-forward NN with three hidden layers by using $\tanh \left(x\right)$ as the activation function was employed to represent a WF. The input layer has 10 nodes, equal to the number of internuclear distances for the molecule. In the loss function in Eq. (7), we use γ=1/50,000 when we use atomic units as the unit for the energy term.

We first construct an initial WF ${\psi }^{{{{\rm{NM}}}}}\left(\vec{{{{\boldsymbol{r}}}}}\right)$ as the direct product of nine normal mode WFs with the antisymmetric stretch in the first excited state, the other eight in the ground state. Then we pretrain the NN by performing a Fit(16200, 45, 45, 5) process defined in the Methods section, followed by a Fit(18600, 45, 45, 5) process to get the initialized NN for training ${\psi }^{0}\left(\vec{{{{\boldsymbol{r}}}}}\right)$ with a fitting error, measured in terms of the root mean square error (RMSE), of ~10^−5^. We have carefully tested various neural network architectures, and found an NN with three hidden layers offers the best overall performance in terms of accuracy and computational efficiency for both the fitting and the subsequent training processes.

**Fig. 1:**
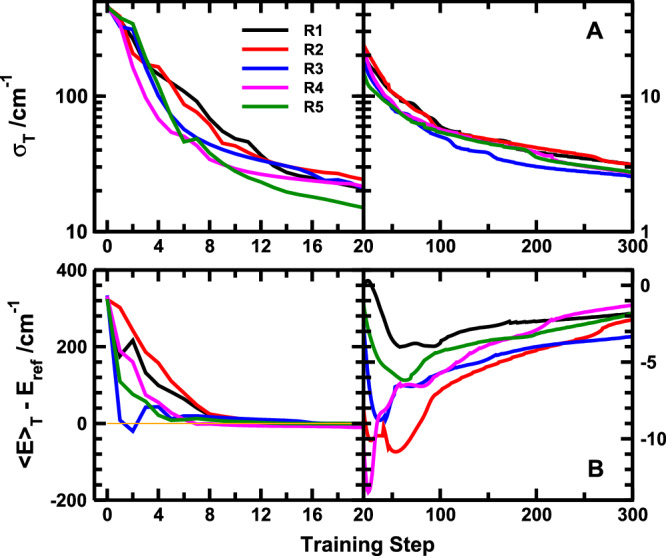
First-round training convergence starting from ${\psi }^{0}\left(\vec{{{{\boldsymbol{r}}}}}\right)$. **A** The training-point standard deviation ${\sigma }_{{{{\rm{T}}}}}$ and (**B**) the average energy ${\left\langle E\right\rangle }_{{{{\rm{T}}}}}$ for 5 replications, labeled R1–R5, as a function of training steps for the first antisymmetric stretch excited state of CH_4_ starting from ${\psi }^{0}\left(\vec{{{{\boldsymbol{r}}}}}\right)$ obtained by fitting normal mode WF. Source data are provided as a Source Data file.

With ${\psi }^{0}\left(\vec{{{{\boldsymbol{r}}}}}\right)$, we carry out a training process Train(18600, 45, 45, 5) defined in the Methods section. Figure 1 shows the evolution of the training-point average energy ${\left\langle E\right\rangle }_{{{{\rm{T}}}}}$ defined in Eq. (5) and the standard deviation ${\sigma }_{{{{\rm{T}}}}}$ defined in Eq. (4), respectively, as training proceeds for 5 replications with a total number of 2801 NN parameters. For all these 5 replications, ${\sigma }_{{{{\rm{T}}}}}$ decreases very quickly from more than 400 cm^−1^ to about 20 cm^−1^ in about 20 training steps, and reduce further to about 3 cm^−1^ after 300 steps (Fig. 1A). Figure 1B shows the differences between ${\left\langle E\right\rangle }_{{{{\rm{T}}}}}$ and the reference energy for the state obtained by the standard variational method using basis functions^67^ for these 5 replications also decrease very quickly from ~300 cm^−1^ to less than 15 cm^−1^ in 20 steps, and further reduces to less than 4 cm^−1^ in 300 steps, demonstrating clearly that ${\left\langle E\right\rangle }_{{{{\rm{T}}}}}$ can converge very rapidly to the desired eigenvalue for the antisymmetric stretch excited state without any orthogonality requirement imposed on the WF as the training proceeds. Because the working points used in this round of training were obtained by sampling the normal mode WF, we stop the training process after 300 steps and save the trained WF as ${\psi }^{1}\left(\vec{{{{\boldsymbol{r}}}}}\right)$ for the next round of training.

**Fig. 2:**
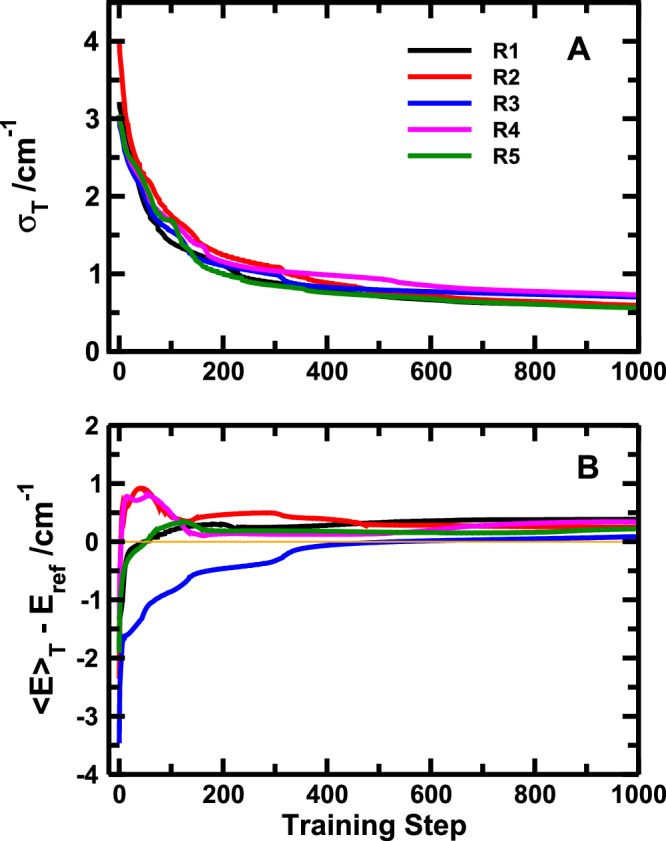
Second-round training convergence starting from ${\psi }^{1}\left(\vec{{{{\boldsymbol{r}}}}}\right)$. **A** The training-point standard deviation ${\sigma }_{{{{\rm{T}}}}}$ and (**B**) the average energy ${\left\langle E\right\rangle }_{{{{\rm{T}}}}}$ for 5 replications, labeled R1–R5, as a function of training steps for the first antisymmetric stretch excited state of CH_4_ by training ${\psi }^{1}\left(\vec{{{{\boldsymbol{r}}}}}\right)$. Source data are provided as a Source Data file.

For the second round of training, we sample ${\psi }^{1}\left(\vec{{{{\boldsymbol{r}}}}}\right)$ and perform a Fit(18100, 45, 45, 5) process, followed by a Fit(20800, 45, 45, 5) process. We then carry out a Train(20800, 45, 45, 5) process to further improve the training accuracy. On the new batch of working points, the initial ${\sigma }_{{{{\rm{T}}}}}$ values for all 5 replications are smaller than 4 cm^−1^ (Fig. 2A). As the training proceeds, ${\sigma }_{{{{\rm{T}}}}}$ decreases steadily to <1 cm^−1^ in about 500 steps, and reduces further to <0.75 cm^−1^ after 1000 steps. The differences between ${\left\langle E\right\rangle }_{{{{\rm{T}}}}}$ and the reference energy are also reduced from a few wave numbers to <0.5 cm^−1^ in about 500 steps and remain more or less unchanged as training proceeds further (Fig. 2B).

Supplementary Table 1 lists the differences between the training-point average energy ${\left\langle E\right\rangle }_{{{{\rm{T}}}}}$ and the reference energy, and the standard deviation ${\sigma }_{{{{\rm{T}}}}}$ at the 500th training step for these 5 replications. For all the replications, ${\sigma }_{{{{\rm{T}}}}}$ is less than 1 cm^−1^, with an average of 0.18 cm^−1^ and a standard deviation of 0.13 cm^−1^, indicating the average of the local energies of the training points can achieve sub-wavenumber accuracy.

Now let us examine the performance of the sample-point averaged energy ${\left\langle E\right\rangle }_{{{{\rm{S}}}}}$ at the 500th training step by sampling points ${\vec{{{{\boldsymbol{r}}}}}}_{i}$ according to the weight ${w}_{i}={\left|\left.{\psi }^{1}({\vec{{{{\boldsymbol{r}}}}}}_{i})\right|\right.}^{2}$. We take ${\psi }^{1}\left(\vec{{{{\boldsymbol{r}}}}}\right)$ for the third replication (R3 as shown in Fig. 2) as an example, and sample it with 5×10^6^ points. Supplementary Figure 2 shows the local energies and the corresponding WF magnitudes for 1/5 of these sample points. No matter for the large magnitude part of the WF (Supplementary Fig. 2A) or the small magnitude part of the WF (Supplementary Fig. 2B), the sample points are distributed evenly around the reference energy. However, the sample points spread substantially in energy as the magnitude of the WF reduces, starting from ~ 1 cm^−1^ at $\left|{\psi }^{1}\left(\vec{{{{\boldsymbol{r}}}}}\right)\right| \sim 0.8$ to a few hundred wavenumbers when $\left|{\psi }^{1}\left(\vec{{{{\boldsymbol{r}}}}}\right)\right| \sim {10}^{-3}$ with a few points even scattered to >2000 cm^−1^ for$\,\left|{\psi }^{1}\left(\vec{{{{\boldsymbol{r}}}}}\right)\right| < {10}^{-3}$.

**Fig. 3:**
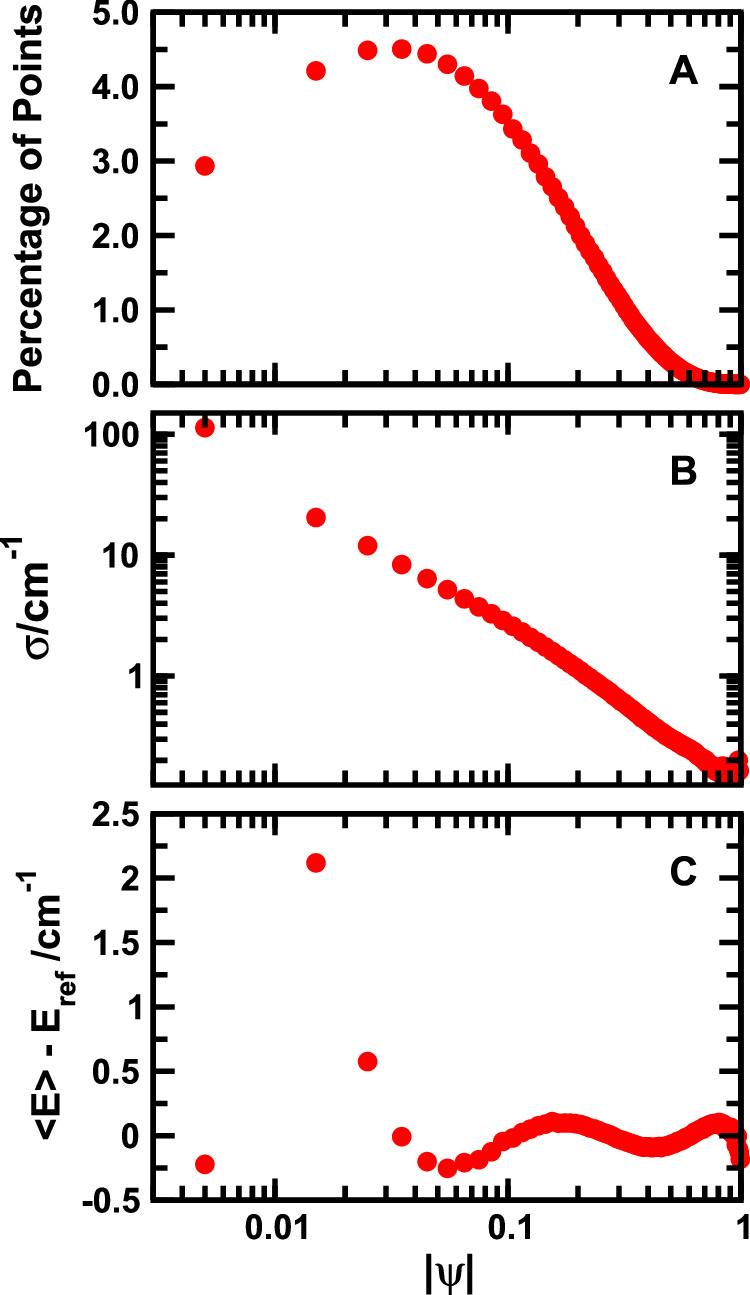
Sampling distribution and local energy statistics across wavefunction amplitude intervals. **A** The percentage of sampling points in each interval divided according to the magnitude of WF. **B** The standard deviation of local energies for the sampling points in each interval. **C** The average of local energies for the sampling points in each interval. Source data are provided as a Source Data file.

To provide a quantitative measurement of the average energy and spread of sample points shown in Supplementary Fig. 3 in different $\left|{\psi }^{1}\left(\vec{{{{\boldsymbol{r}}}}}\right)\right|$ regions, we divide the magnitude of $\left|{\psi }^{1}\left(\vec{{{{\boldsymbol{r}}}}}\right)\right|$ into 100 intervals and calculate the average and standard deviation of the sampling points in each interval. Figure 3A shows the percentage of sampling points in each interval. It turns out the intervals between 0.01 and 0.06 have high percentages larger than 4%. There are about 3% of the sampling points with $\left|{\psi }^{1}\left(\vec{{{{\boldsymbol{r}}}}}\right)\right| < 0.01$.

Figure 3B depicts the standard deviation of local energies for the sampling points, ${\sigma }_{{{{\rm{s}}}}}$, in these intervals. ${\sigma }_{{{{\rm{s}}}}}$ is very small in the intervals with large $\left|{\psi }^{1}\left(\vec{{{{\boldsymbol{r}}}}}\right)\right|$, and increases steadily with the decrease of $\left|{\psi }^{1}\left(\vec{{{{\boldsymbol{r}}}}}\right)\right|$, reaching 113 cm^−1^ in the last interval of $({{\mathrm{0.0,0.01}}})$. The overall ${\sigma }_{{{{\rm{s}}}}}$ is 20.3 cm^−1^, apparently due to the large deviations in the small $\left|{\psi }^{1}\left(\vec{{{{\boldsymbol{r}}}}}\right)\right|$ region.

In contrast, the average of local energies for the sampling points in all the intervals only varies slightly around the reference energy as shown in Fig. 3C. The differences between the average energies and the reference energy are smaller than 0.3 cm^−1^, except in the $({{\mathrm{0.01,0.02}}})$ and $({{\mathrm{0.02,0.03}}})$ intervals, which have differences of 2.1 cm^−1^ and 0.57 cm^−1^, respectively.

Supplementary Table 1 also shows ${\left\langle E\right\rangle }_{{{{\rm{s}}}}}-{E}_{{{{\rm{ref}}}}}$ and ${\sigma }_{{{{\rm{s}}}}}$ for the 5 replications at the 500th training step. For all the replications, ${\sigma }_{{{{\rm{s}}}}}$ is between 10 and 21 cm^−1^, substantially larger than ${\sigma }_{{{{\rm{T}}}}}$. However, ${\left\langle E\right\rangle }_{{{{\rm{s}}}}}-{E}_{{{{\rm{ref}}}}}$ is smaller than ${\left\langle E\right\rangle }_{{{{\rm{T}}}}}-{E}_{{{{\rm{ref}}}}}$ except the 3rd replication, with an average of 0.066 cm^−1^ and a standard deviation of 0.028 cm^−1^, indicating the average energies on the sampling points can also achieve sub-wavenumber accuracy with 500 training steps. The outcome that ${\left\langle E\right\rangle }_{{{{\rm{s}}}}}$ is more accurate than ${\left\langle E\right\rangle }_{{{{\rm{T}}}}}$ is very appealing, and is even a bit beyond our expectations. The overall behavior of the sampling-point average energy, i.e., very accurate ${\left\langle E\right\rangle }_{{{{\rm{s}}}}}$ with quite large ${\sigma }_{{{{\rm{s}}}}}$, is similar to diffusion MC calculations in which rather large energy fluctuation always occurs in an individual simulation, but the average energy is very accurate for the ground state^48^. By increasing the training step to 1000, we can see from Supplementary Table 2 that the average energies and the standard deviations for both training points and sampling points only scarcely change, indicating 500 training steps are sufficient.

To illustrate the evolution of the WF in training, we show in Supplementary Fig. 3 some one-dimensional cuts of the trained WFs at the first 20 training steps as a function of one of the CH distances. The one-dimensional cut changes very quickly from the normal mode to the final WF in merely 20 steps, revealing clearly that we are able to train the WF rapidly towards the true eigenfunction (see SI for more details).

**Fig. 4:**
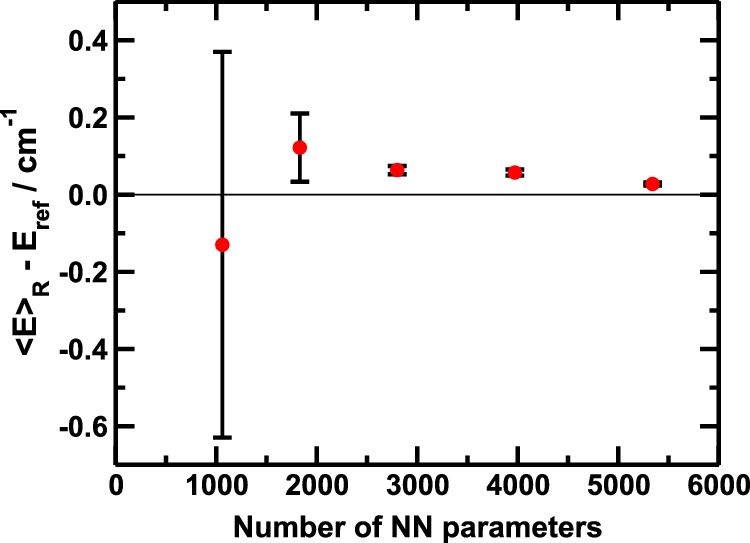
Neural-network size dependence of vibrational energy accuracy. The replication-averaged energy 〈E〉_R_ as a function of the number of parameters for the first antisymmetric stretch excited state of CH_4_. Error bars denote the corresponding standard deviations, ${\sigma }_{{{{\rm{R}}}}}$. Source data are provided as a Source Data file.

Figure 4 shows the replication-averaged energy ${\left\langle E\right\rangle }_{{{{\rm{R}}}}}$ and its corresponding standard deviation ${\sigma }_{{{{\rm{R}}}}}$ as a function of NN parameters. By merely using 1061 (10,25,25,5,1) NN parameters, we are able to obtain a rather accurate ${\left\langle E\right\rangle }_{{{{\rm{R}}}}}$ that is only lower than the reference energy by 0.13 cm^−1^, albeit with a fairly large ${\sigma }_{R}$ of ~0.5 cm^−1^, which is adequately accurate for most applications. With the increase of NN parameters, both the energy difference and ${\sigma }_{{{{\rm{R}}}}}$ decrease quickly, in particular ${\sigma }_{{{{\rm{R}}}}}$. For 5341 (10,65,65,5,1) NN parameters, ${\left\langle E\right\rangle }_{{{{\rm{R}}}}}$ = 0.028 cm^–1^ and ${\sigma }_{{{{\rm{R}}}}}$ = 0.0038 cm^–1^.

In Table 1, we show ${\left\langle E\right\rangle }_{{{{\rm{R}}}}}$ and ${\sigma }_{{{{\rm{R}}}}}$ for the 7 selected vibrational states of the CH_4_ molecule, including the ground state, four fundamental excited states, one overtone (0200) state, and one combined (1100) state, in terms of both frequency and level energy. Those results were obtained from 5 replications by training ${\psi }^{1}\left(\vec{{{{\boldsymbol{r}}}}}\right)$ with 500 training steps, using 2801 (10,45,45,5,1) parameters for the four fundamental excited states, and 4631 (10,60,60,5,1) parameters for the overtone (0200) and combined (1100) states. The difference between ${\left\langle E\right\rangle }_{{{{\rm{R}}}}}$ and the reference value is <0.1 cm^−1^ except for the symmetric stretch (1000) state of 0.42 cm^−1^ and the overtone (0200) state of −0.41 cm^−1^.

**Table 1:** The replication-averaged energies 〈E〉_R_, frequency Δ〈E〉_R_ and the standard deviation (${\sigma }_{{{{\rm{R}}}}}$) (in units of cm^–1^) for the 7 selected vibrational states of the CH_4_ molecule obtained from five replications, in comparison with the results from variational calculations^67^

State	E_ref_	∆E_ref_	〈E〉_R_	∆〈E〉_R_	〈E〉_R_-E_ref_	σ_R_
0000	9651.293	0.000	9651.315	0.000	0.022	0.0125
0001	10961.764	1310.471	10961.775	1310.460	0.011	0.0037
0100	11184.756	1533.463	11184.781	1533.466	0.025	0.0082
1000	12568.464	2917.171	12568.889	2917.574	0.425	0.2438
0010	12670.726	3019.433	12670.792	3019.477	0.066	0.0277
0200	12716.594	3065.301	12716.206	3064.891	-0.388	0.2861
1100	14087.263	4435.970	14087.362	4436.047	0.099	0.0338

Supplementary Table 3 shows the overlap matrix for all the CH_4_ vibrational states that are listed in Table 1. The diagonal elements of this matrix indicate that these WFs are normalized. Most off-diagonal elements are on the order of 10^−4^, and some of them are even smaller than 10^−4^. The largest overlap element is −8.4 × 10^−3^ for the (1000) and (0200) states. Therefore, all the calculated WFs for the vibrational states are indeed orthogonal within numerical uncertainty as expected.

Supplementary Figs. 4 and 5 show one-dimensional cuts of the wavefunctions along each of the four CH bonds in CH₄ for the ground and first symmetric CH stretching excited state, respectively. As seen in these figures, all four cuts are identical for these two symmetric states. We also show in Supplementary Figs. 6 and 7, the values of the wave functions at 1000 Monte Carlo sampled configurations before and after the permutation of two hydrogen atoms. Similarly, the wave functions remain unchanged after permutation. Therefore, although the permutational symmetry was not enforced in our calculations, the obtained wavefunctions exhibit the proper symmetry.

To show further the obtained wave functions can be used to calculate accurate expectation values, we list the average values of the four CH bond lengths in Supplementary Table 4 and their standard deviations in Supplementary Table 5 for the ground and four fundamental excited states, in comparison with those from the variational method^68^. First, one can see that the four average values of CH bond lengths and their standard deviations for all these states are both identical to the fourth decimal place, further verifying the permutation symmetry of the calculated wave functions. They are also identical to the variational results to the fourth decimal place, indicating the calculated wave functions using NN method are of high accuracy. In Supplementary Tables 6 and 7, we show the average values and the standard deviations for six H–C–H angles for these five states. As for the CH bond lengths in Supplementary Tables 4 and 5, all the average values and the standard deviations obtained from the NN wave functions agree perfectly with each other, and they also agree very well those from the variation calculation.

To demonstrate the capability of our NN method, we apply it to the propane (C_3_H_8_) molecule, a system with 11 atoms and 27 degrees of freedom, on an accurate potential energy surface constructed very recently by our group^69^. The feed-forward NN with three hidden layers is employed to represent the wave function. The input layer has 55 nodes, equal to the number of internuclear distances for a molecular configuration.

For the ground state, we first construct ${\psi }^{{{{\rm{NM}}}}}\left(\vec{{{{\boldsymbol{r}}}}}\right)$ as a direct product of 27 normal mode wave functions in the ground state. Then we perform a Fit(100,000, 90, 90, 15) process to get ${\psi }^{0}\left(\vec{{{{\boldsymbol{r}}}}}\right)$, followed by a Train(100,000, 90, 90, 15) process. Suppl. Fig. 8A shows the evolution of the standard deviation ${\sigma }_{{{{\rm{T}}}}}$ as a function of training steps for C_3_H_8_ with the weight parameter in Eq. (4) equal to 2. Similar to CH_4_ training, for all 5 replications, ${\sigma }_{{{{\rm{T}}}}}$ decreases very quickly from ~230 cm^−1^ to less than 10 cm^–1^ in about 100 training steps, and reduces further to less than 2 cm^–1^ after 1000 steps. Suppl. Fig. 8B shows that the differences between ${\left\langle E\right\rangle }_{{{{\rm{T}}}}}$ for these 5 replications and the reference energy for the state obtained by the diffusion Monte Carlo method also decrease very quickly from more than 300 cm^–1^ to about 10 cm^–1^ in 100 steps, and further reduce to less than 4 cm^–1^ in 1000 steps. (See the SI for details of the DMC calculation.) Then we perform two additional 1,000-step training processes, gradually adding more training points to reach totals of 116,000 and 132,000, respectively.

Supplementary Table 8 shows the training and sampling energies for the five replications after 3,000 training steps, together with their corresponding standard deviations. Although the training energies ${\left\langle E\right\rangle }_{{{{\rm{T}}}}}$ are higher than the DMC value by more than 2 cm^–1^, the sampling energies ${\left\langle E\right\rangle }_{{{{\rm{S}}}}}$ differ from the DMC value by <0.1 cm^–1^, with the sample standard deviations ${\sigma }_{{{{\rm{s}}}}}$ between 20 and 30 cm^–1^. To reduce the standard deviations ${\sigma }_{{{{\rm{s}}}}}$ further, we perform an additional 1000 steps training with a weight parameter equal to 1.5. As can be seen from Supplementary Table 9, the sampling standard deviations are reduced by about 10 cm^–1^, and the differences between the training energies and the DMC value drop to <1.1 cm^–1^, while the differences between the sampling energies and the DMC value remain below 0.1 cm^–1^.

We perform the same procedure as that used for the ground state for three vibrationally excited states involving C-C-C bonds, i.e. C-C-C deformation, symmetric and antisymmetric stretches, using the same NN parameters but with 10,000 additional training points. In Table 2, we show the replication-averaged energies and the standard deviations for the ground and three vibrationally excited states of the C_3_H_8_ molecule obtained from 5 replications. As can be seen, whether for the ground state or the excited states, the replication standard deviations are <0.2 cm^–1^, indicating the sampling energies are very well converged with a total of 4000 training steps. For the ground state, the replication-averaged energy is 22398.56 cm^–1^, in perfect agreement with the DMC value of 22398.62±0.49 cm^–1^. For the three vibrationally excited states, the replication-averaged energies agree very well with the corresponding experimental values within the experimental uncertainties, showing that our NN-based method is capable of yielding vibrational energies with spectroscopic accuracy for C_3_H_8_^70,71^. Note that we only used fewer than 150,000 training points and employed an NN with 14,400 parameters for C_3_H_8_, an 11-atom molecule with 27 degrees of freedom; this clearly demonstrates the remarkable efficiency of our NN-based method in solving the high-dimensional SE.

**Table 2:** The replication-averaged energies (${\left\langle E\right\rangle }_{{{{\rm{R}}}}}$) and the standard deviation (${\sigma }_{{{{\rm{R}}}}}$) (in units of cm^–1^) for the ground state and three vibrationally excited states of the C_3_H_8_ molecule obtained from five replications, relative to the DMC value of 22398.62 ± 0.49 cm^–1^ for the ground state

State	${\left\langle E\right\rangle }_{{{{\rm{R}}}}}$	${\sigma }_{{{{\rm{R}}}}}$	${{{\rm{E}}}}{{{\rm{xp}}}}.$	${\left\langle E\right\rangle }_{{{{\rm{R}}}}}-{{{\rm{Exp}}}}.$
Ground state	-0.060	0.033		
CCC deformation	367.794	0.065	369 (±3 ~ 6)	-1.2
CCC symmetric stretch	868.209	0.092	869 (±3 ~ 6)	-0.79
CCC antisymmetric stretch	1050.12	0.20	1054 (±3 ~ 6)	−3.9

The experimental frequency is from refs. ^70,71^.

Therefore, we developed a highly efficient NN method to solve the vibrational SEs for polyatomic molecular systems by optimizing the variance of local energies calculated on a batch of molecular configurations selected based on the training errors, and demonstrated that it is capable of providing vibrational energies for C_3_H_8_ with an accuracy of ~1 cm^–1^ using fewer than 15,000 NN parameters. It is conceivable that the proposed NN method can be readily applied to calculate vibrational energies for rigid molecules with >20 atoms. Preliminary studies also show the method is capable of providing accurate vibrational energy levels for molecular clusters, such as (H_2_O)_2_ and (H_2_O)_3_. We will carry out more calculations for both semirigid and fluxional systems, such as those provided in VIBFREQ1295^72^, V30^73^, and Barone et al.^74^ in follow-up studies. Because our approach requires a global potential energy surface for each system, we will need to construct high-quality PESs if they are not available.

Finally, we note that the Hamiltonian in Eq. (6) is for the total molecular angular momentum J=0, and all calculations presented in this work are for vibrational energy levels. In principle, one can use the rovibrational Hamiltonian in Eq. (3) of the SI to compute rovibrational energies. However, the inclusion of the rotation requires higher computational precision due to the small spacing of rotational energy levels, especially for larger molecules. Therefore, calculating accurate rovibrational levels is considerably more challenging and will require further effort.

## Methods

For a given SE, $\hat{H}\psi \left(\vec{{{{\boldsymbol{r}}}}}\right)=\left(\hat{T}+V\left(\vec{{{{\boldsymbol{r}}}}}\right)\right)\psi \left(\vec{{{{\boldsymbol{r}}}}}\right)=E\psi \left(\vec{{{{\boldsymbol{r}}}}}\right)$, the eigenfunction $\psi \left(\vec{{{{\boldsymbol{r}}}}}\right)$ must satisfy this equation throughout the entire configuration space. Defining the local energy at a configuration point ${\vec{{{{\boldsymbol{r}}}}}}_{i}\,$(where $\psi \left(\vec{{{{\boldsymbol{r}}}}}\right)\ne 0$) as

3 $${E}_{{{{\rm{l}}}}}\left({\vec{{{{\boldsymbol{r}}}}}}_{i}\right)=\frac{\hat{T}\psi ({\vec{{{{\boldsymbol{r}}}}}}_{i})}{\psi \left({\vec{{{{\boldsymbol{r}}}}}}_{i}\right)}+V\left({\vec{{{{\boldsymbol{r}}}}}}_{i}\right)$$

we note that ${E}_{{{{\rm{l}}}}}\left({\vec{{{{\boldsymbol{r}}}}}}_{i}\right)$ is a constant for the exact solution, whereas for an approximate wave function it varies with configuration position. We exploit this property to optimize the NN parameters by using the variance of the local energies over a set of training configurations as the loss function for NN training,

4 $${\sigma }_{{{{\rm{T}}}}}^{2}=\frac{\mathop{\sum }_{i=1}^{N}{\left({E}_{{{{\rm{l}}}}}\left({\vec{{{{\boldsymbol{r}}}}}}_{i}\right)-{\left\langle E\right\rangle }_{{{{\rm{T}}}}}\right)}^{2}{w}_{i}}{\mathop{\sum }_{i=1}^{N}{w}_{i}}$$

where N is the number of configuration points,$\,{w}_{i}$ is the weight for each point, and ${\left\langle E\right\rangle }_{{{{\rm{T}}}}}$ is the average of the local energies over the training set with weight ${w}_{i}$,

5 $${\left\langle E\right\rangle }_{{{{\rm{T}}}}}=\frac{\mathop{\sum }_{i=1}^{N}{{E}_{{{{\rm{l}}}}}\left({\vec{{{{\boldsymbol{r}}}}}}_{i}\right)w}_{i}}{\mathop{\sum }_{i=1}^{N}{w}_{i}}$$

When we choose ${w}_{i}={\left|\left.\psi \left({\vec{{{{\boldsymbol{r}}}}}}_{i}\right)\right|\right.}^{2}$, Eq. (4) looks exactly the same as Eq. (1), but they are very different in the summation. For Eq. (1), the summation runs over quadrature points or MC sampling points designed to converge the configuration space integral. In Eq. (4), however, the points are chosen deliberately based on training needs. This allows us to select configuration points where the local energy exhibits large deviations from its average to effectively reduce training errors in the configuration space. Consequently, as we will demonstrate, we can use a small number of points in Eq. (4) to achieve small local energy deviations across the important regions of the configuration space.

It is worth noting that the selection of arbitrary point sets in training is similar in spirit to that of the collocation method, which was introduced by Yang and Peet into chemical community using the same number of basis functions and collocation points^75^. Later, Manzhos and Carrington proposed the rectangular collocation method by using more collocation points than the basis functions^56,64,76–80^. These collocation points can be randomly sampled in the configuration space where the wave functions are nonzero to achieve higher efficiency in calculations.

When we use internuclear distances as input molecular coordinates for a WF, the space-fixed Cartesian KEO for the total angular momentum J=0 for a molecule with an arbitrary number of atoms can be simply written as

6 $$\hat{T}=\mathop{\sum }_{i=1}^{{N}_{a}}-\frac{{\hslash }^{2}}{2{m}_{i}}{\nabla }^{2}=\mathop{\sum}_{i=1}^{{N}_{a}}-\frac{{\hslash }^{2}}{2{m}_{i}}\left[{\sum}_{\,\,j=1,j\ne i}^{{N}_{a}}\,{\sum}_{k=1,k\ne i}^{{N}_{a}}\cos \left({\theta }_{{jik}}\right)\frac{{\partial }^{2}}{\partial {r}_{{ij}}\partial {r}_{{ik}}}+{\sum}_{j=1,j\ne i}^{{N}_{a}}\frac{2}{{r}_{{ij}}}\frac{\partial }{\partial {r}_{{ij}}}\right]$$

where ${N}_{a}$ is the number of atoms in the molecular system, ${r}_{{ij}}$ is the internuclear distance between the *i*th and *j*th atoms, and ${\theta }_{{jik}}$ is the angle between the ${r}_{{ij}}$ and ${r}_{{ik}}$ bonds (see SI for detailed derivation). We note that Manzhos and Carrington used the space-fixed Cartesian KEO to compute the kinetic energy of a WF that depends on internal curvilinear coordinates^81^. Our approach follows the same spirit, but we use internuclear distances instead of internal curvilinear coordinates to describe a molecular configuration.

Our iterative procedure for solving the SE by using NN is composed of two tasks in every iteration: WF fitting and WF training. In a WF fitting, we use an NN to fit a given WF $\psi \left(\vec{{{{\boldsymbol{r}}}}}\right)$ to a desired accuracy as in PES fitting^82–89^ (see SI for details). We denote a WF fitting process that uses *N* fitting points and a neural network with architecture of $({n}_{0},{n}_{1},{n}_{2},\cdots,{n}_{l},1)$ as Fit(*N*, ${n}_{1}$, ${n}_{2},\cdots,{n}_{l}$). Here ${n}_{0}$ is the number of internuclear distances in a molecular system, l is the number of hidden layers, and ${n}_{i}\,(i=1,\cdots,l)$ denotes the number of neurons in the i-th hidden layer.

In WF training, we employ the Levenberg-Marquardt algorithm^90^ to optimize NN parameters. To avoid getting the trivial solution for the WF, we add a penalty term in the loss function given in Eq. (4) by fixing the value of the WF at one molecular configuration at its initial value provided by the fitting process,

7 $$\,{{{\boldsymbol{L}}}}={\sigma }_{{{{\rm{T}}}}}^{2}+\gamma {\left(\psi \left({\vec{{{{\boldsymbol{r}}}}}}_{{{{\bf{0}}}}}\right)-{\psi }_{0}\left({\vec{{{{\boldsymbol{r}}}}}}_{{{{\bf{0}}}}}\right)\right)}^{2}$$

where$\,{\vec{{{{\boldsymbol{r}}}}}}_{{{{\boldsymbol{0}}}}}$ is a molecular configuration with relatively large WF amplitude, and ${\psi }_{0}\left({\vec{{{{\boldsymbol{r}}}}}}_{{{{\boldsymbol{0}}}}}\right)$ is the initial value of the WF at ${\vec{{{{\boldsymbol{r}}}}}}_{{{{\boldsymbol{0}}}}}$. We start a training process using a set of training points with$\,{w}_{i}={\left|\left.\psi \left({\vec{{{{\boldsymbol{r}}}}}}_{i}\right)\right|\right.}^{2}$. When the learning rate becomes low, we can stop the training process, sample the trained WF, select some configurations with large training errors relative to the average energy, add them to the training data set, and repeat the training. This process to increase training points may be repeat a few times until the loss function cannot be reduced substantially further. Analogously to Fit(*N*, ${n}_{1}$, ${n}_{2},\cdots,{n}_{l}$), we denote a WF training process that uses *N* training points and a neural network with architecture of $({n}_{0},{n}_{1},{n}_{2},\cdots,{n}_{l},1)$ as Train(*N*, ${n}_{1}$, ${n}_{2},\cdots,{n}_{l}$).

We use a normal mode WF for the ground state or one of the excited states, which can be obtained easily, as our initial WF ${\psi }^{{{{\rm{NM}}}}}\left(\vec{{{{\boldsymbol{r}}}}}\right)$. As in early studies on solving the SE for harmonic oscillators^36^, we write the WF as a product of Gaussian functions and an NN function to assure correct asymptotic behavior for bound states (see SI for the details for the Gaussian functions). We first carry out WF fitting to fit ${\psi }^{{{{\rm{NM}}}}}\left(\vec{{{{\boldsymbol{r}}}}}\right)$ to obtain ${\psi }^{0}\left(\vec{{{{\boldsymbol{r}}}}}\right)$ expressed by an NN. We then train ${\psi }^{0}\left(\vec{{{{\boldsymbol{r}}}}}\right)$ using the data points from the WF fitting to obtain ${\psi }^{1}\left(\vec{{{{\boldsymbol{r}}}}}\right)$. If needed, ${\psi }^{1}\left(\vec{{{{\boldsymbol{r}}}}}\right)$ can be further refined by iteratively adding points with large training errors to the training set.

Because the normal mode WF is normally rather different from the true WF in the configuration space, we can sample the trained WF ${\psi }^{1}\left(\vec{{{{\boldsymbol{r}}}}}\right)$, which is expected to be closer to the true WF, fit and train ${\psi }^{1}\left(\vec{{{{\boldsymbol{r}}}}}\right)$ with an increasing number of training points to get ${\psi }^{2}\left(\vec{{{{\boldsymbol{r}}}}}\right)$. If necessary, we can carry out the procedure iteratively to obtain ${\psi }^{n}\left(\vec{{{{\boldsymbol{r}}}}}\right)$ with increasingly more working points.

For every ${\psi }^{n}\left(\vec{{{{\boldsymbol{r}}}}}\right)$ we obtain from WF training, we sample it with a weight of ${\left|\left.{\psi }^{n}\left(\vec{{{{\boldsymbol{r}}}}}\right)\right|\right.}^{2}$ and calculate the sample-point averaged energy ${\left\langle E\right\rangle }_{{{{\rm{S}}}}}$ according to Eq. (2) by using a large number of sample points. Because ${\left\langle E\right\rangle }_{{{{\rm{S}}}}}$ depends on the initial NN parameters and training points, to assess the accuracy of ${\left\langle E\right\rangle }_{{{{\rm{S}}}}}$, we run a few replications of the training process to obtain the average and standard error of ${\left\langle E\right\rangle }_{{{{\rm{S}}}}}$, which are denoted as ${\left\langle E\right\rangle }_{{{{\rm{R}}}}}$ and ${\sigma }_{{{{\rm{R}}}}}$. When the replication standard error ${\sigma }_{{{{\rm{R}}}}}$ is converged to a desired accuracy, we stop the training process for this set of NN parameters. Finally, we may vary the number of NN parameters to check if ${\left\langle E\right\rangle }_{{{{\rm{R}}}}}$ is converged with respect to the number of NN parameters. For clarity, we depict in Supplementary Fig. 1 the flowchart of our NN approach to solve the time-independent SE for molecular vibrational states.

## Supplementary information

Supplementary Information

Transparent Peer Review file

## Source data

Source Data

## Data Availability

The data generated and used in this study, as well as the PES files are available on Zenodo under accession code 10.5281/zenodo.20537455. Source data are provided with this paper.

## Code availability

The source code used in this work to pretrain and train neural networks, evaluate the vibrational energies, and perform the diffusion Monte Carlo calculations is publicly available on Zenodo under accession code 10.5281/zenodo.20537455. The code is distributed under the MIT License.
