# Supplementary material for: Solving the vibrational Schrödinger equation with artificial neural networks
Source: Nat Commun. 2026 Jun 20;17:7789. doi: 10.1038/s41467-026-74537-4 (PMC13438559; doi:10.1038/s41467-026-74537-4)
Supplement: Supplementary file 1 [file 41467_2026_74537_MOESM1_ESM.pdf]

Supplementary Information for "Solving the vibrational Schrödinger equation with artificial neural networks"

Shuaishuai Zhao[1,2] & Dong H. Zhang[1,3,*]

1. State Key Laboratory of Chemical Reaction Dynamics, Dalian Institute of Chemical Physics, Chinese Academy of Sciences, Dalian, 116023, China.
2. University of Chinese Academy of Sciences, Beijing, 100049, China.
3. Hefei National Laboratory, Hefei, 230088, China

Suppl. Note 1. Derivation of the Kinetic Energy Operator

In a space-fixed Cartesian coordinate system, using atomic units, the vibrational kinetic energy operator for an N_a-atom molecule is given by:

$$\hat{T} = -\sum_{i=1}^{N_a}\sum_{\beta=1}^{3}\frac{1}{2m_i}\frac{\partial^2}{\partial\left(x_i^{(\beta)}\right)^2} \tag{1}$$

where m_i is the mass of atom i, and $x_i^{(\beta)}$ (for $\beta = 1,2,3$) represent the three Cartesian coordinate components of atom i.

By choosing three center-of-mass (CM) coordinates, three Euler angles, and internal configurational coordinates to describe the molecule in space, the kinetic energy operator can be rewritten as follows[1,2]:

$$\hat{T} = \hat{T}^{\mathrm{CM}} + \sum_{ij} A_{ij}\hat{p}_i\hat{p}_j + \sum_i B_i\hat{p}_i + \sum_{mn} C_{mn}\hat{J}_m\hat{J}_n + \sum_m D_m\hat{J}_m + \sum_{im} E_{im}\hat{p}_i\hat{J}_m \tag{2}$$

where A, B, C, D and E are coordinate-dependent matrices, $\hat{p}_i = \frac{\partial}{\partial q_i}$, and $\hat{T}^{\mathrm{CM}}$ is the center-of-mass kinetic energy operator. Since the potential energy surface is independent of the molecular CM coordinates, the translational energy is not considered when solving for the vibrational states. Thus, the CM kinetic energy operator $\hat{T}^{\mathrm{CM}}$ can be omitted:

$$\hat{T} = \sum_{ij} A_{ij}\hat{p}_i\hat{p}_j + \sum_i B_i\hat{p}_i + \sum_{mn} C_{mn}\hat{J}_m\hat{J}_n + \sum_m D_m\hat{J}_m + \sum_{im} E_{im}\hat{p}_i\hat{J}_m \tag{3}$$

Next, we examine the form of the kinetic energy operator when the total angular momentum quantum number is zero ($J = 0$). Letting $|\varphi\rangle$ denote the quantum state

with $J = 0$, the action of the angular momentum operators on this state ($|\varphi\rangle$) yields zero. Consequently, all terms in the KEO involving these operators vanish:

$$\sum_{mn} C_{mn}\hat{J}_m\hat{J}_n|\varphi\rangle + \sum_{m} D_m\hat{J}_m|\varphi\rangle + \sum_{im} E_{im}\hat{p}_i\hat{J}_m|\varphi\rangle = 0 \tag{4}$$

Therefore, for the zero angular momentum case ($J = 0$), one can express the vibrational KEO using only the internal coordinates via the coordinate transformation from Cartesian to internal coordinates.

$$\hat{T} = -\sum_{i=1}^{N_a}\sum_{\beta=1}^{3}\frac{1}{2m_i}\frac{\partial^2}{\partial\left(x_i^{(\beta)}\right)^2} = \sum_{ij} A_{ij}\hat{p}_i\hat{p}_j + \sum_{i} B_i\hat{p}_i \tag{5}$$

Then, we derive the explicit expression for the kinetic energy operator at $J = 0$ in terms of a specific set of internal coordinates. We choose all pairwise interatomic distances as the internal coordinates, defined by the set $\{r_{ij}|i = 1,2,3 \dots N_a - 1, j = i+1, i+2, \dots, N_a\}$:

$$r_{ij} = |\,\vec{x}_i - \vec{x}_j| = \sqrt{\sum_{\beta=1}^{3}\left(x_i^{(\beta)} - x_j^{(\beta)}\right)^2} \tag{6}$$

The derivative of r_{ij} with respect to $x_i^{(\beta)}$ is:

$$\frac{\partial r_{ij}}{\partial x_i^{(\beta)}} = \frac{1}{r_{ij}}\left(x_i^{(\beta)} - x_j^{(\beta)}\right) \tag{7}$$

Using the chain rule, we can get the first derivative operator and the second derivative operator, which can be expressed as:

$$\frac{\partial}{\partial x_i^{(\beta)}} = \sum_{j=1,j\neq i}^{N_a}\left(\frac{\partial r_{ij}}{\partial x_i^{(\beta)}}\frac{\partial}{\partial r_{ij}}\right) = \sum_{j=1,j\neq i}^{N_a}\left(x_i^{(\beta)} - x_j^{(\beta)}\right)\frac{1}{r_{ij}}\frac{\partial}{\partial r_{ij}} \tag{8}$$

$$\frac{\partial^2}{\partial\left(x_i^{(\beta)}\right)^2} = \sum_{j=1,j\neq i}^{N_a}\left\{\left[\frac{\partial}{\partial x_i^{(\beta)}}\left(\frac{1}{r_{ij}}\frac{\partial}{\partial r_{ij}}\right)\right]\left(x_i^{(\beta)} - x_j^{(\beta)}\right) + \frac{1}{r_{ij}}\frac{\partial}{\partial r_{ij}}\right\} \tag{9}$$

Further expanding the expression for the second derivative, we can obtain:

$$\frac{\partial^2}{\partial\left(x_i^{(\beta)}\right)^2} = \sum_{j=1,j\neq i}^{N_a}\sum_{k=1,k\neq i}^{N_a}\left(x_i^{(\beta)} - x_j^{(\beta)}\right)\left(x_i^{(\beta)} - x_k^{(\beta)}\right)\frac{1}{r_{ij}r_{ik}}\frac{\partial^2}{\partial r_{ij}\partial r_{ik}} + \sum_{j=1,j\neq i}^{N_a} -\left(x_i^{(\beta)} - x_j^{(\beta)}\right)^2\frac{1}{r_{ij}^3}\frac{\partial}{\partial r_{ij}} + \frac{1}{r_{ij}}\frac{\partial}{\partial r_{ij}} \tag{10}$$

Substituting Eq. (10) into Eq. (1) yields the kinetic energy operator expression with the interatomic distances as molecular configuration coordinates when $J = 0$.

$$\begin{aligned}\hat{T} &= \sum_{i=1}^{N_a}\frac{-1}{2m_i}\nabla_i^2 \\ &= \sum_{i=1}^{N_a}\left[\frac{-1}{2m_i}\sum_{j=1,j\neq i}^{N_a}\sum_{k=1,k\neq i}^{N_a} cos(\theta_{jik})\frac{\partial^2}{\partial r_{ij}\partial r_{ik}}\right] + \sum_{i=1}^{N_a}\left[\frac{-1}{2m_i}\sum_{j=1,j\neq i}^{N_a} 2\frac{1}{r_{ij}}\frac{\partial}{\partial r_{ij}}\right]\end{aligned} \tag{11}$$

where θ_{jik} is the angle formed by the internuclear vectors r_{ij} and r_{ik}.

Suppl. Note 2. Gaussian Function

In this work, the wavefunction ($\psi_{\mathrm{NN}}(r_1, r_2, \dots, r_M)$) is expressed as a product of Gaussian functions and an NN function,

$$\psi_{\mathrm{NN}}(r_1, r_2, \dots, r_M) = g_j(r_1, r_2, \dots, r_M) f_{\mathrm{NN}}(r_1, r_2, \dots, r_M) \tag{12}$$

where r_i is the i-th internuclear distance, and $M = N_a(N_a - 1)/2$ is the number of all internuclear distances (N_a is the number of atoms). The term $f_{\mathrm{NN}}(r_1, r_2, \dots, r_M)$ represents a fully connected feedforward neural network with an input layer of M nodes. The functions $g_1(r_1, r_2, \dots, r_M)$ and $g_2(r_1, r_2, \dots, r_M)$ are two types of Gaussian functions used in this work, with $j = 1,2$.

For the ground state and the four fundamental excited states of methane, the first type of Gaussian function is used, which is the product of all the Gaussian functions with respect to a single internuclear distance,

$$g_1(r_1, r_2, r_3, \dots, r_M) = \exp\left(\sum_{i=1}^{M} -\frac{(r_i - \mu_i)^2}{2\sigma_i^2}\right) \tag{13}$$

Here, μ_i and σ_i represent the center and the standard deviation of the Gaussian function, respectively.

The second type of Gaussian function is used in the wavefunctions of the overtone (0200) and combination (1100) states of methane and all vibrational states of propane. It is constructed as the product of $(3N_a - 6)$ Gaussian functions with respect to $(3N_a - 6)$ linearly transformed coordinates of the internuclear distances,

$$g_2(r_1, r_2, r_3, \dots, r_M) = \exp\left(\sum_{i=1}^{3N-6} -\frac{\left(\sum_{j=1}^{M} C_{ij}(r_j - \mu_j)\right)^2}{2\sigma_i^2}\right) \quad (14)$$

where C is the linear transformation matrix, and μ_j is the internuclear distance of the reference configuration.

All corresponding parameters are provided in the attachment "parameters of Gaussian function.txt". For the first type of Gaussian function, there are two lines of parameters corresponding to the center (μ_i) and the standard deviation (σ_i). For the second type of Gaussian function, the first and second lines correspond to the internuclear distance of the reference configuration (μ_j) and the standard deviation (σ_i), respectively. The linear transformation matrix (C) is presented below the standard deviation, and it is separated by a line reading 'c matrix'.

Suppl. Note 3. Computational Details for Diffusion Monte Carlo (DMC) Calculations

To accurately compute the ground-state zero-point energy (ZPE) of propane, we implemented a standard unguided diffusion Monte Carlo algorithm[3-5] using 3N Cartesian coordinates and performed the DMC calculations nine times with the same running parameters. The imaginary time step was 2.0 a.u., and the population control parameter was 0.25. In each simulation, the number of initial walkers was 1,000,000, and the total number of diffusion steps was 1,010,000, of which the first 5,000 steps were treated as the burn-in steps for equilibration. The ZPE of propane (22398.62 cm^{-1}) and its statistical uncertainty (0.49 cm^{-1}) were obtained as the average and the standard deviation of the reference energies from the 9 DMC calculations, respectively.

Suppl. Note 4. Wave Function Fitting

In a WF fitting, we use a NN to fit a given WF $\psi(\vec{r})$ (divided by a Gaussian function) to a desired accuracy as in PES fitting[6-13]. Because the value of $\psi(\vec{r})$ at any molecular configuration can be evaluated with negligible computational cost, we can directly compare the fitted WF with $\psi(\vec{r})$ to select the configurations with large fitting errors to achieve high fitting accuracy easily. We first use MC method to sample $|\psi(\vec{r})|^2$ to obtain a large number of molecular sampling configurations. Out of these MC sampling configurations we randomly select N points to perform a few NN fittings (~ 5 times) of $\psi(\vec{r})$ with different initial NN parameters. We then evaluate the values of the fitted wave functions at all these MC points (except those N points), and select another ΔN points with largest fitting error relative to $\psi(\vec{r})$. Using $N + \Delta N$ points we perform another ~5 NN fittings and select the wave function with the least fitting error as one initial NN wave function for WF training.

Suppl. Note 5. Evolution of Wave Function

To illustrate the evolution of the WF in training, we show in Suppl. Fig. 3 some one-dimensional cuts of the trained WFs at the first 20 training steps as a function of one of the CH distances with other three CH bond lengths and all the bond angles fixed at a geometry with maximum WF value. We set the maximum value of the WF at 1 for clarity. As can be seen, the positions of maximum and minimum of the initial normal mode WF are away from their final positions by 0.04 a_0 (upper insert) and 0.07 a_0 (lower insert), respectively. In the first step, the peak of trained WF shifts to the right by 0.013 a_0, while the valley becomes much deeper with the position of bottom shifted to the right by ~ 0.01 a_0. In the second step, the position of the maximum continues to move to the right, and depth of the valley reduce considerably. Somehow, in the fifth step, the peak moves to the left slightly and becomes to overlap that for the second step, and the valley becomes deeper than the second step. From then on, the position of the peak shifts to the right all the way, the depth of the valley is reduced steadily, respectively, to their final values. The one-dimensional cut of the trained WF becomes to be identical to the true WF after 20 steps, revealing clearly that we are able to train the WF rapidly

towards the true eigenfunction.

Suppl. Table 1. Training-point average energy $\langle E \rangle_{\mathrm{T}}$, sample-point average energy $\langle E \rangle_{\mathrm{S}}$, and their corresponding standard deviations for the first antisymmetric stretch excited state by training $\psi^1(\vec{\boldsymbol{r}})$ for 500 steps for 5 replications. E_{ref}=12670.726 cm^{-1} (Ref. 14)

Replication	$\langle E \rangle_{\mathrm{T}} - E_{\mathrm{ref}}$	σ_{T}	$\langle E \rangle_{\mathrm{S}} - E_{\mathrm{ref}}$	σ_{s}
1	0.3286	0.7133	0.0390	11.7572
2	0.2923	0.7746	0.0434	13.8764
3	-0.0098	0.7933	0.0812	20.2964
4	0.1368	0.9345	0.1054	17.3901
5	0.1652	0.7154	0.0598	20.7772
Average	0.1826		0.0658	
Standard deviation	0.1349		0.0277	

Suppl. Table 2. Same as Suppl. Table 1 except by training $\psi^1(\vec{\boldsymbol{r}})$ for 1000 steps.

Replication	$\langle E\rangle_{\mathrm{T}} - E_{\mathrm{ref}}$	σ_{T}	$\langle E\rangle_{\mathrm{S}} - E_{\mathrm{ref}}$	σ_{S}
1	0.3869	0.5854	0.0347	10.7458
2	0.2501	0.5952	0.0347	13.7275
3	0.0899	0.6984	0.0658	19.3959
4	0.3416	0.7281	0.0652	15.4440
5	0.2211	0.5617	0.0590	16.4617
Average	0.2579		0.0519	
Standard deviation	0.1154		0.0159	

Suppl. Table 3. The overlap matrix for all the CH_4 vibrational states listed in Table I. The numbers in parentheses denote powers of 10; for example 2.82(-4) represents 2.82×10^{-4}.

	0000	0001	0100	1000	0010	0200	1100
0000	1.00	-1.02(-4)	2.82(-4)	3.19(-4)	4.19(-4)	-2.81(-4)	9.42(-5)
0001		1.00	-4.23(-5)	9.53(-5)	2.62(-4)	-4.59(-4)	-3.51(-4)
0100			1.00	4.09(-4)	2.79(-4)	-1.31(-5)	5.04(-5)
1000				1.00	-2.36(-3)	-8.38(-3)	2.60(-4)
0010					1.00	-1.32(-3)	9.47(-5)
0200						1.00	5.27(-5)
1100							1.00

Suppl. Table 4. Expectation values ($\langle r_{CH} \rangle$) of the four C-H bond lengths (r_{CH}) of the CH_4 molecule in the vibrational ground state and the four fundamental excited states (in units of Bohr). Reference values are obtained from standard variational calculations[15]. The numbers in parentheses denote powers of 10.

State		$\langle r_{CH} \rangle$			
0000	Ref	2.0921	2.0921	2.0922	2.0921
	This work	2.0923	2.0922	2.0923	2.0922
	Error	1.5(-4)	7.2(-5)	9.2(-5)	6.6(-5)
0001	Ref	2.0946	2.0955	2.0959	2.0958
	This work	2.0945	2.0953	2.0958	2.0958
	Error	-1.2(-4)	-1.4(-4)	-9.9(-5)	-5.5(-6)
0100	Ref	2.0960	2.0960	2.0960	2.0960
	This work	2.0959	2.0961	2.0960	2.0959
	Error	-3.5(-5)	1.5(-4)	6.8(-5)	-2.7(-5)
1000	Ref	2.1069	2.1069	2.1069	2.1069
	This work	2.1067	2.1070	2.1068	2.1070
	Error	-2.6(-4)	3.0(-5)	-7.6(-5)	3.4(-5)
0010	Ref	2.1253	2.1251	2.0913	2.0913
	This work	2.1252	2.1251	2.0915	2.0913
	Error	-6.8(-5)	7.3(-6)	1.7(-4)	3.7(-5)

Suppl. Table 5. The standard deviation values ($\sigma_{r_{CH}}$) of the four C-H bond lengths (r_{CH}) of the CH_4 molecule in the vibrational ground state and the four fundamental excited states (in units of Bohr). Reference values as in Suppl. Table 4. The numbers in parentheses denote powers of 10.

State		$\sigma_{r_{CH}}$			
0000	Ref	0.1465	0.1465	0.1464	0.1465
	This work	0.1464	0.1464	0.1465	0.1465
	Error	-1.9(-5)	-1.8(-5)	3.9(-5)	1.8(-6)
0001	Ref	0.1472	0.1469	0.1468	0.1468
	This work	0.1472	0.1469	0.1468	0.1468
	Error	-2.1(-5)	5.1(-6)	2.3(-5)	2.2(-5)
0100	Ref	0.1469	0.1469	0.1469	0.1469
	This work	0.1469	0.1470	0.1469	0.1468
	Error	1.4(-6)	7.6(-5)	1.3(-5)	-3.8(-5)
1000	Ref	0.1791	0.1791	0.1788	0.1791
	This work	0.1789	0.1792	0.1792	0.1793
	Error	-2.2(-4)	9.5(-5)	3.9(-4)	2.5(-4)
0010	Ref	0.2096	0.2094	0.1463	0.1464
	This work	0.2095	0.2094	0.1465	0.1465
	Error	-7.0(-5)	2.8(-6)	1.7(-4)	1.4(-4)

Suppl. Table 6. Expectation values ($\langle\theta_{\mathrm{HCH}}\rangle$) of the six HCH bond angles (θ_{HCH}) of the CH_4 molecule in the vibrational ground state and the four fundamental excited states (in units of degrees). Reference values as in Suppl. Table 4. The numbers in parentheses denote powers of 10.

State		$\langle\theta_{\mathrm{HCH}}\rangle$					
0000	Ref	109.169	109.168	109.168	109.168	109.168	109.167
	This work	109.177	109.174	109.177	109.154	109.154	109.173
	Error	7.9(-3)	5.6(-3)	8.4(-3)	-1.4(-2)	-1.3(-2)	5.4(-3)
0001	Ref	108.689	109.004	108.908	108.966	109.055	108.787
	This work	108.658	108.987	108.888	108.987	109.092	108.793
	Error	-3.1(-2)	-1.7(-2)	-2.0(-2)	2.0(-2)	3.7(-2)	6.5(-3)
0100	Ref	109.301	109.218	109.252	109.254	109.219	109.300
	This work	109.260	109.230	109.278	109.282	109.240	109.251
	Error	-4.1(-2)	1.1(-2)	2.6(-2)	2.9(-2)	2.1(-2)	-4.8(-2)
1000	Ref	109.154	109.152	109.152	109.151	109.151	109.149
	This work	109.160	109.139	109.148	109.155	109.148	109.162
	Error	6.3(-3)	-1.3(-2)	-3.8(-3)	3.9(-3)	-3.3(-3)	1.3(-2)
0010	Ref	108.547	109.165	109.200	109.258	109.230	109.526
	This work	108.550	109.205	109.226	109.228	109.195	109.524
	Error	2.9(-3)	4.0(-2)	2.6(-2)	-3.0(-2)	-3.5(-2)	-1.9(-3)

Suppl. Table 7. The standard deviation values ($\sigma_{\theta_{\mathrm{HCH}}}$) of the six HCH bond angles (θ_{HCH}) of the CH_4 molecule in the vibrational ground state and the four fundamental excited states (in units of degrees). Reference values as in Suppl. Table 4. The numbers in parentheses denote powers of 10.

State		$\sigma_{\theta_{\mathrm{HCH}}}$					
0000	Ref	8.435	8.435	8.435	8.435	8.435	8.435
	This work	8.431	8.431	8.431	8.431	8.431	8.438
	Error	-4.1(-3)	-4.6(-3)	-4.3(-3)	-4.3(-3)	-4.4(-3)	2.9(-3)
0001	Ref	8.815	10.729	10.159	10.136	10.710	8.760
	This work	8.773	10.784	10.146	10.122	10.758	8.726
	Error	-4.1(-2)	5.5(-2)	-1.4(-2)	-1.4(-2)	4.8(-2)	-3.3(-2)
0100	Ref	11.268	8.733	9.873	9.873	8.733	11.268
	This work	11.266	8.721	9.894	9.884	8.729	11.267
	Error	-1.9(-3)	-1.1(-2)	2.1(-2)	1.1(-2)	-4.2(-3)	-2.0(-4)
1000	Ref	8.605	8.605	8.605	8.606	8.606	8.606
	This work	8.584	8.584	8.590	8.593	8.579	8.583
	Error	-2.1(-2)	-2.2(-2)	-1.5(-2)	-1.3(-2)	-2.7(-2)	-2.3(-2)
0010	Ref	8.561	8.636	8.657	8.649	8.627	8.451
	This work	8.557	8.621	8.656	8.634	8.627	8.451
	Error	-3.9(-3)	-1.5(-2)	-1.7(-3)	-1.5(-2)	-8.5(-5)	-3.7(-4)

Suppl. Table 8. Training-point average energy $\langle E \rangle_{\mathrm{T}}$, sample-point average energy $\langle E \rangle_{\mathrm{S}}$, and their corresponding standard deviations for the ground state of C_3H_8 by training $\psi^0(\vec{\boldsymbol{r}})$ for 3,000 steps for 5 replications with the weight parameter in Eq. (4) of the main text equal to 2. E_{DMC}=22398.62±0.49 cm^{-1}

Replication	$\langle E \rangle_{\mathrm{T}} - E_{\mathrm{DMC}}$	σ_{T}	$\langle E \rangle_{\mathrm{S}} - E_{\mathrm{DMC}}$	σ_{S}
1	2.20	1.47	0.084	32.0
2	2.11	1.40	-0.013	32.3
3	2.81	1.45	-0.013	37.0
4	2.71	1.41	0.0041	34.8
5	2.29	1.53	0.022	33.8
Average	2.42		0.017	
Standard deviation	0.31		0.040	

Suppl. Table 9. Training-point average energy $\langle E \rangle_{\mathrm{T}}$, sample-point average energy $\langle E \rangle_{\mathrm{S}}$, and their corresponding standard deviations for the ground state of C_3H_8 by training $\psi^0(\vec{\boldsymbol{r}})$ for an additional 1,000 steps for 5 replications with the weight parameter in Eq. (4) of the main text equal to 1.5. E_{DMC}=22398.62±0.49 cm^{-1}

Replication	$\langle E \rangle_{\mathrm{T}} - E_{\mathrm{DMC}}$	σ_{T}	$\langle E \rangle_{\mathrm{S}} - E_{\mathrm{DMC}}$	σ_{S}
1	0.65	1.84	-0.068	24.9
2	0.74	1.76	-0.042	26.8
3	1.07	1.78	-0.018	26.3
4	0.87	1.73	-0.060	24.0
5	0.60	1.77	-0.107	24.1
Average	0.79		-0.0590	
Standard deviation	0.1894		0.0331	

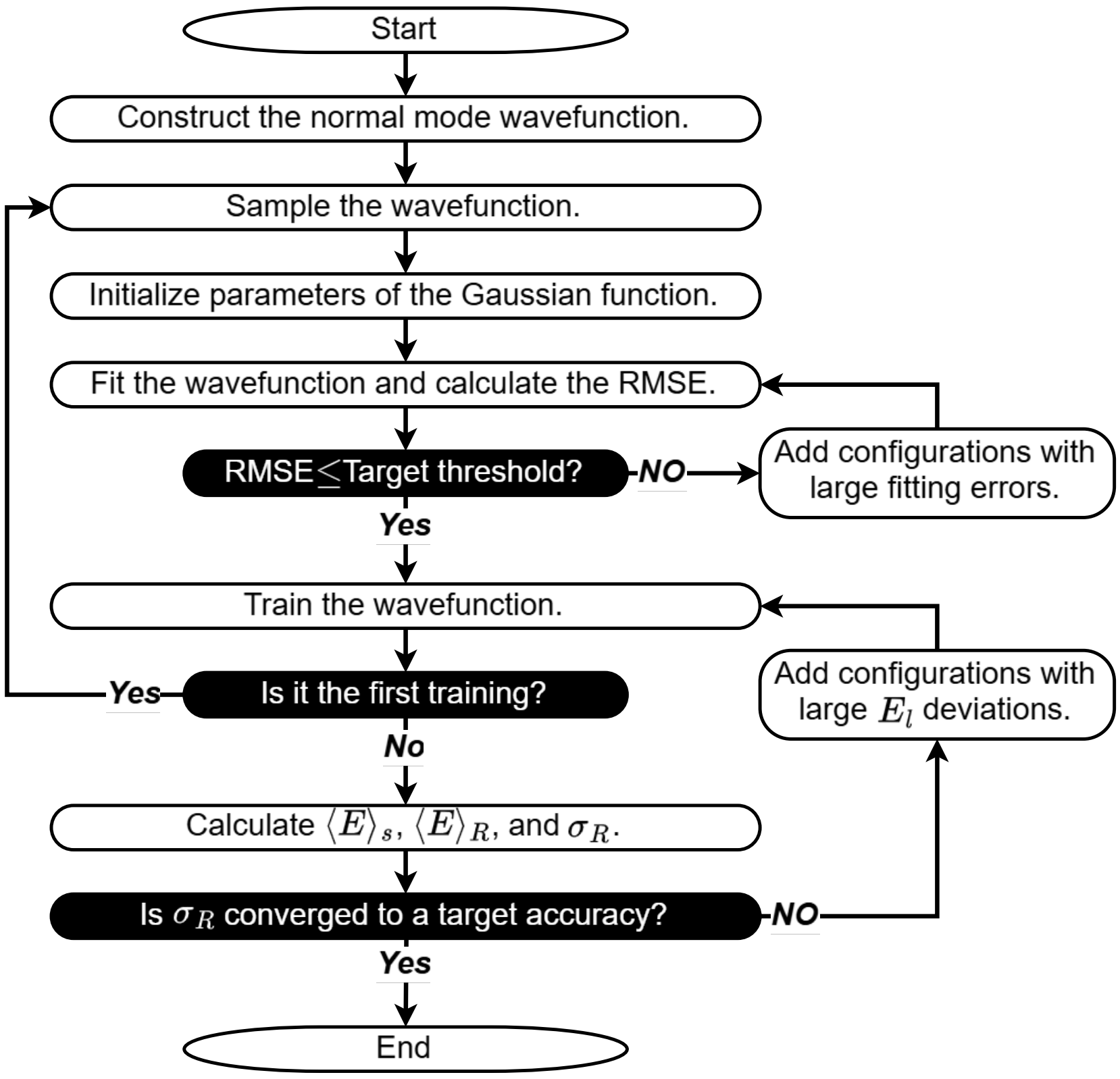

Suppl. Fig. 1. Flowchart of the proposed neural network (NN) approach for solving the vibrational Schrödinger equation.

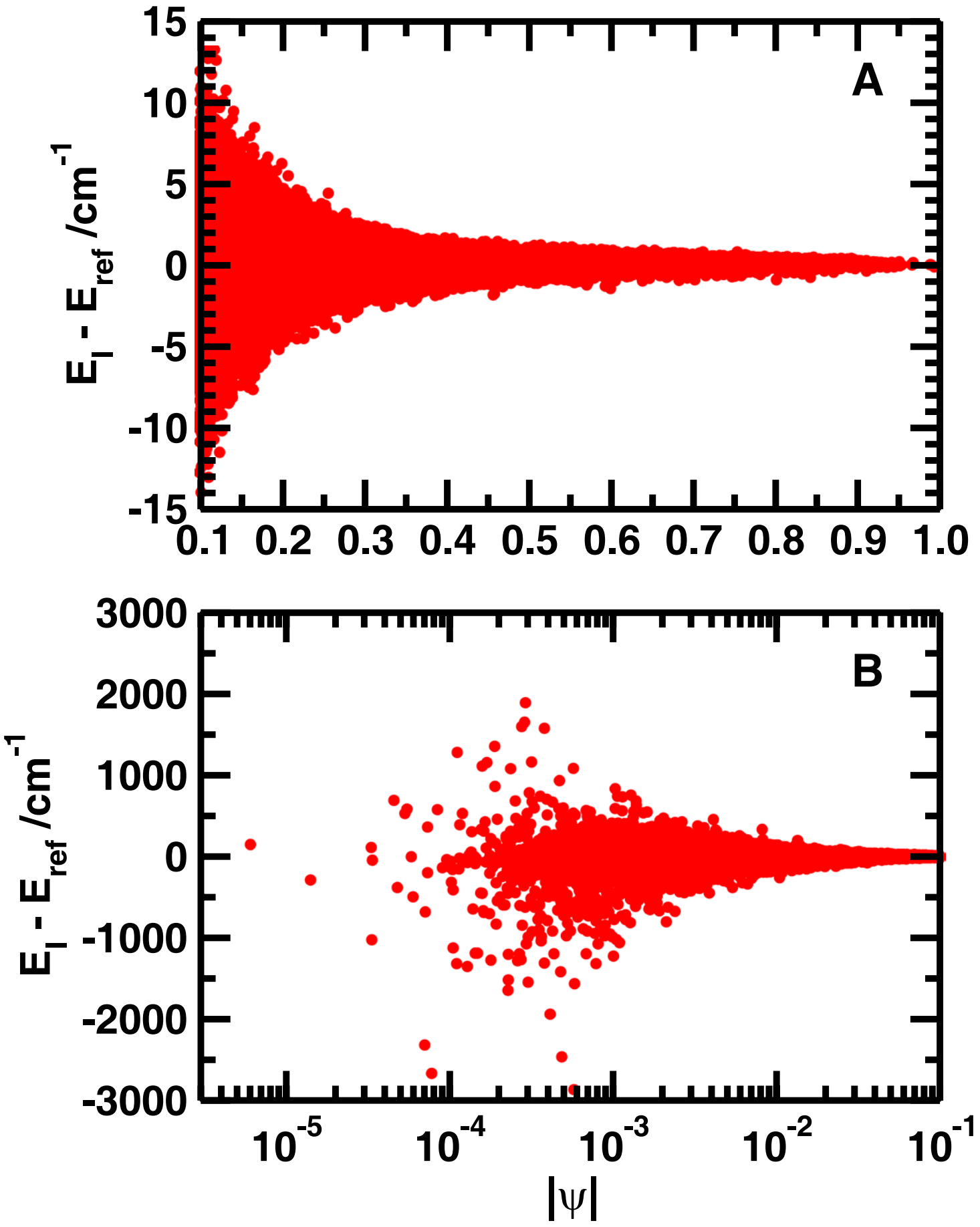

Suppl. Fig. 2. The local energies and the corresponding WF magnitudes for 10^6 sample points for the first antisymmetric stretching excited state of CH_4 by training $\psi^1(\vec{r})$ for 500 steps. Panels A and B show the points with WF magnitudes greater than 0.1 and less than 0.1, respectively. Source data are provided as a Source Data file.

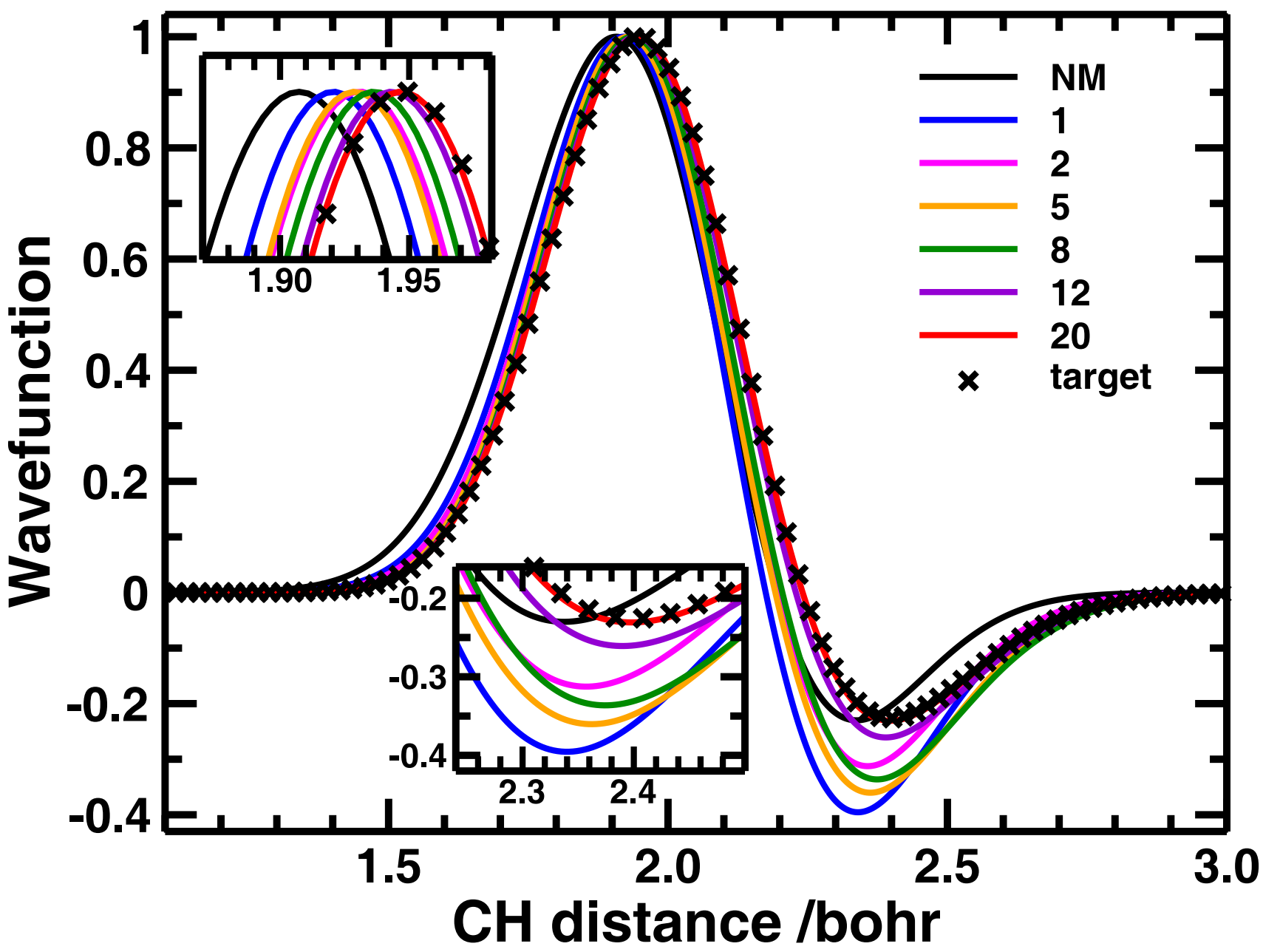

Suppl. Fig. 3. One-dimensional cuts of the trained WFs for the first antisymmetric CH stretching excited state of CH_4 starting from the normal mode WF at the first 20 training steps as a function of CH distance. To show the large-amplitude regions of the WF more clearly, the upper-left and lower insets enlarge the regions near the WF maximum and minimum, respectively. Source data are provided as a Source Data file.

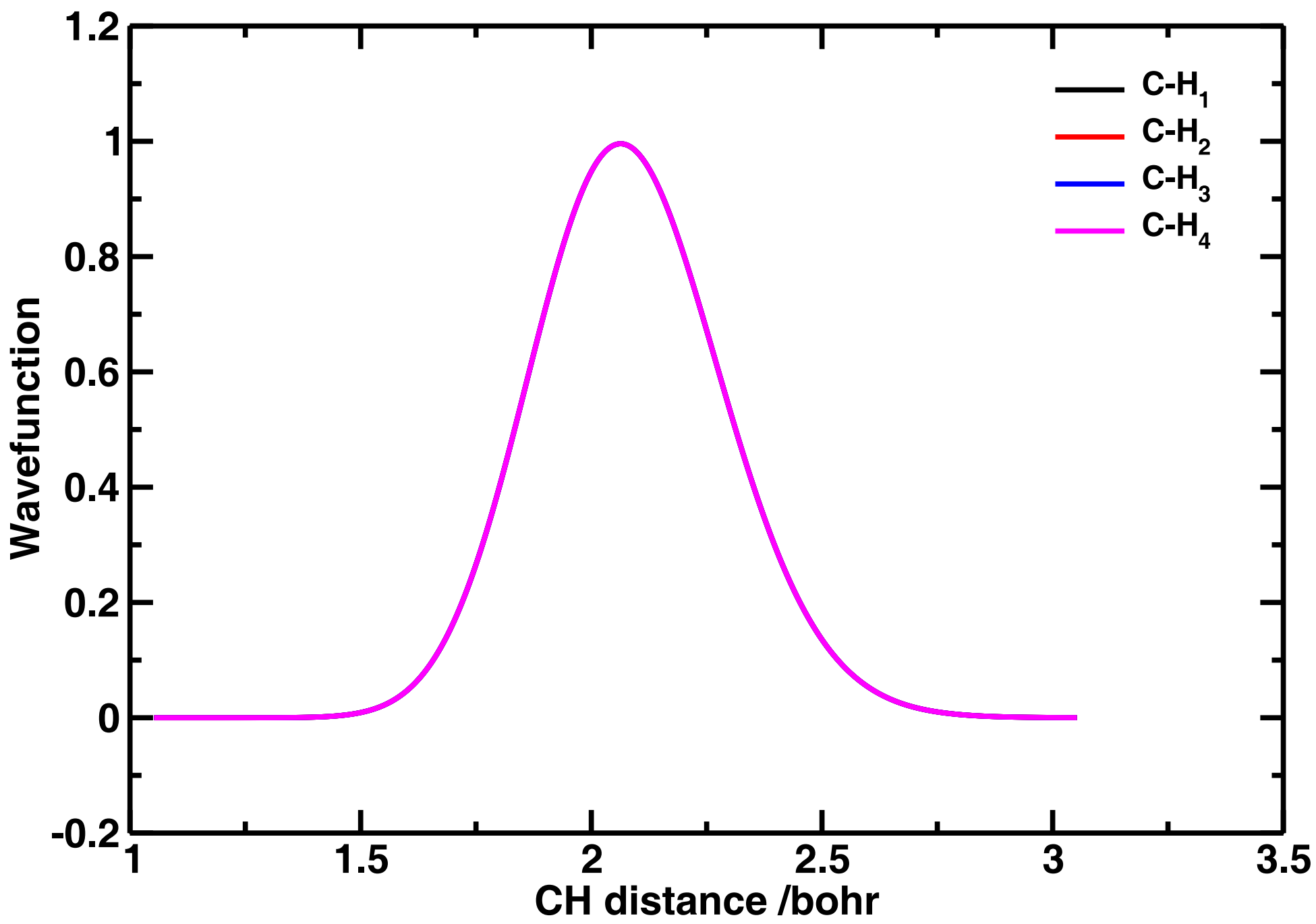

Suppl. Fig. 4. One-dimensional cuts of the trained WFs for the ground state of the CH_4 molecule as a function of CH distances for the four equivalent CH bonds corresponding to $C\text{-}H_1$, $C\text{-}H_2$, $C\text{-}H_3$, and $C\text{-}H_4$. Source data are provided as a Source Data file.

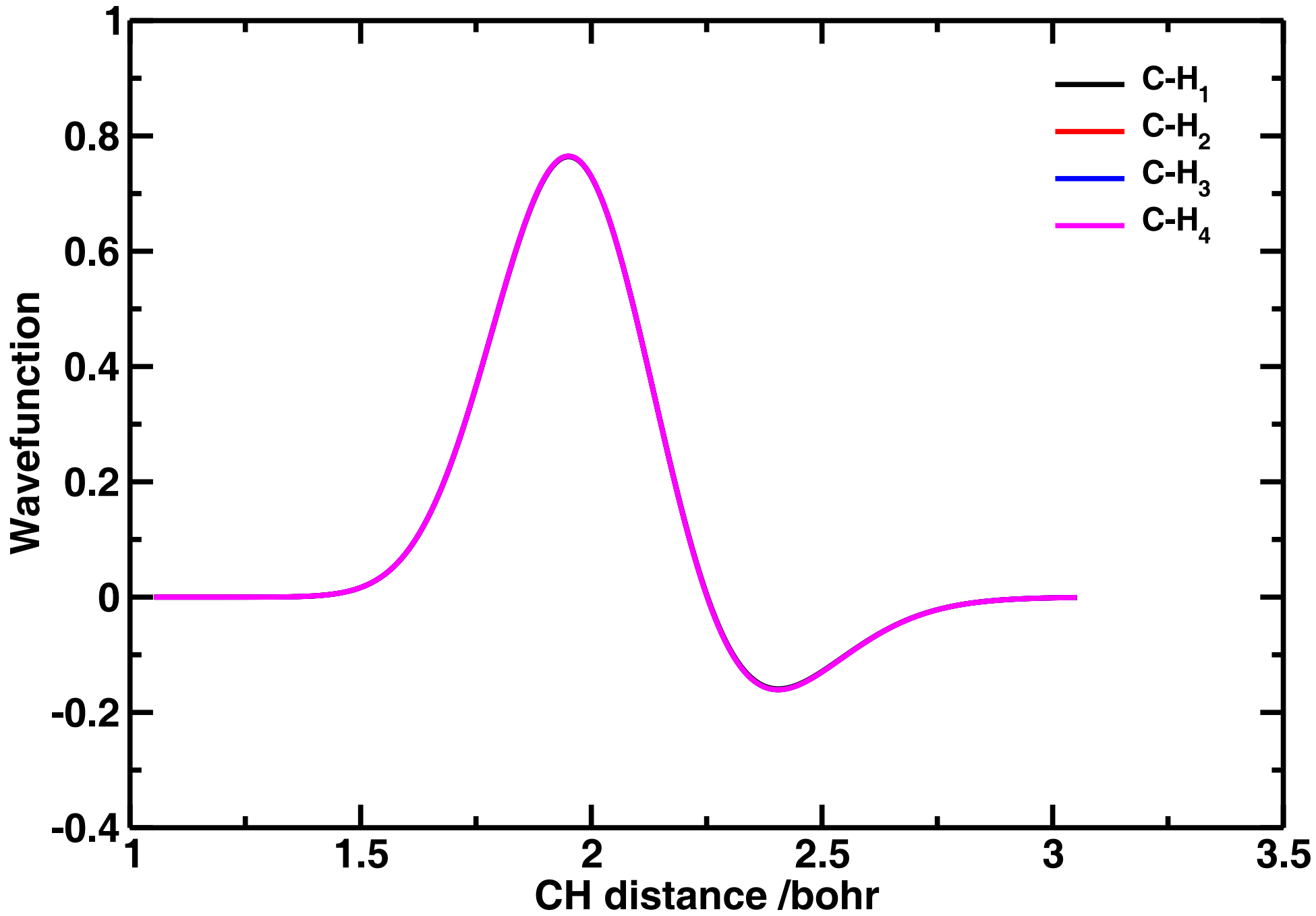

Suppl. Fig. 5. One-dimensional cuts of the trained WFs for the first symmetric C-H stretching excited state of the CH_4 molecule as a function of CH distances for the four equivalent CH bonds corresponding to C-H_1, C-H_2, C-H_3, and C-H_4. Source data are provided as a Source Data file.

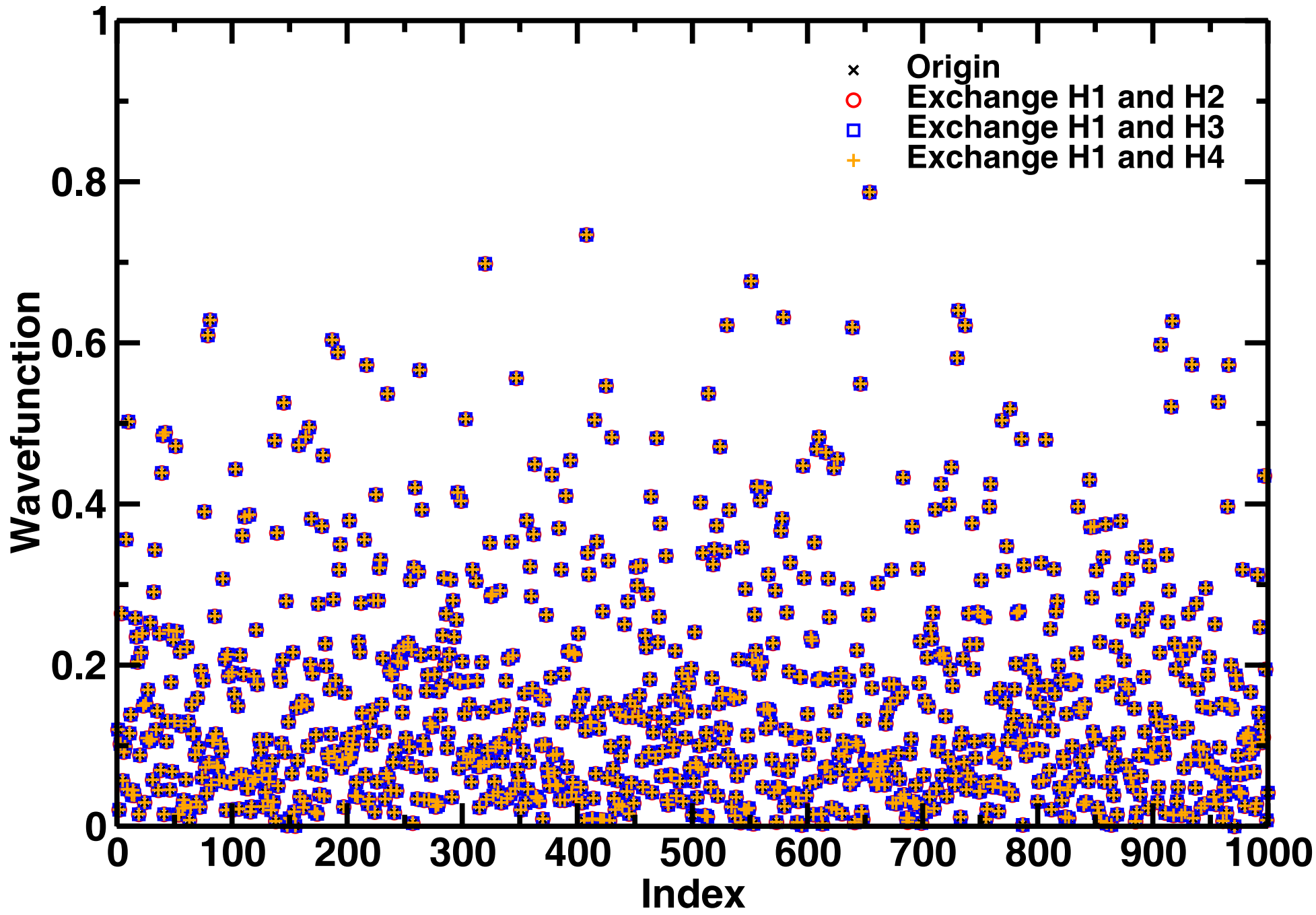

Suppl. Fig. 6. The ground state WFs values evaluated at 1000 MC-sampled configurations of the CH_4 molecule are labeled by black crosses (Origin). The red circles (Exchange H1 and H2), blue squares (Exchange H1 and H3), and orange pluses (Exchange H1 and H4) represent the WF values evaluated at configurations obtained by exchanging the first H atom with the second, the third, and the fourth H atoms, respectively. Source data are provided as a Source Data file.

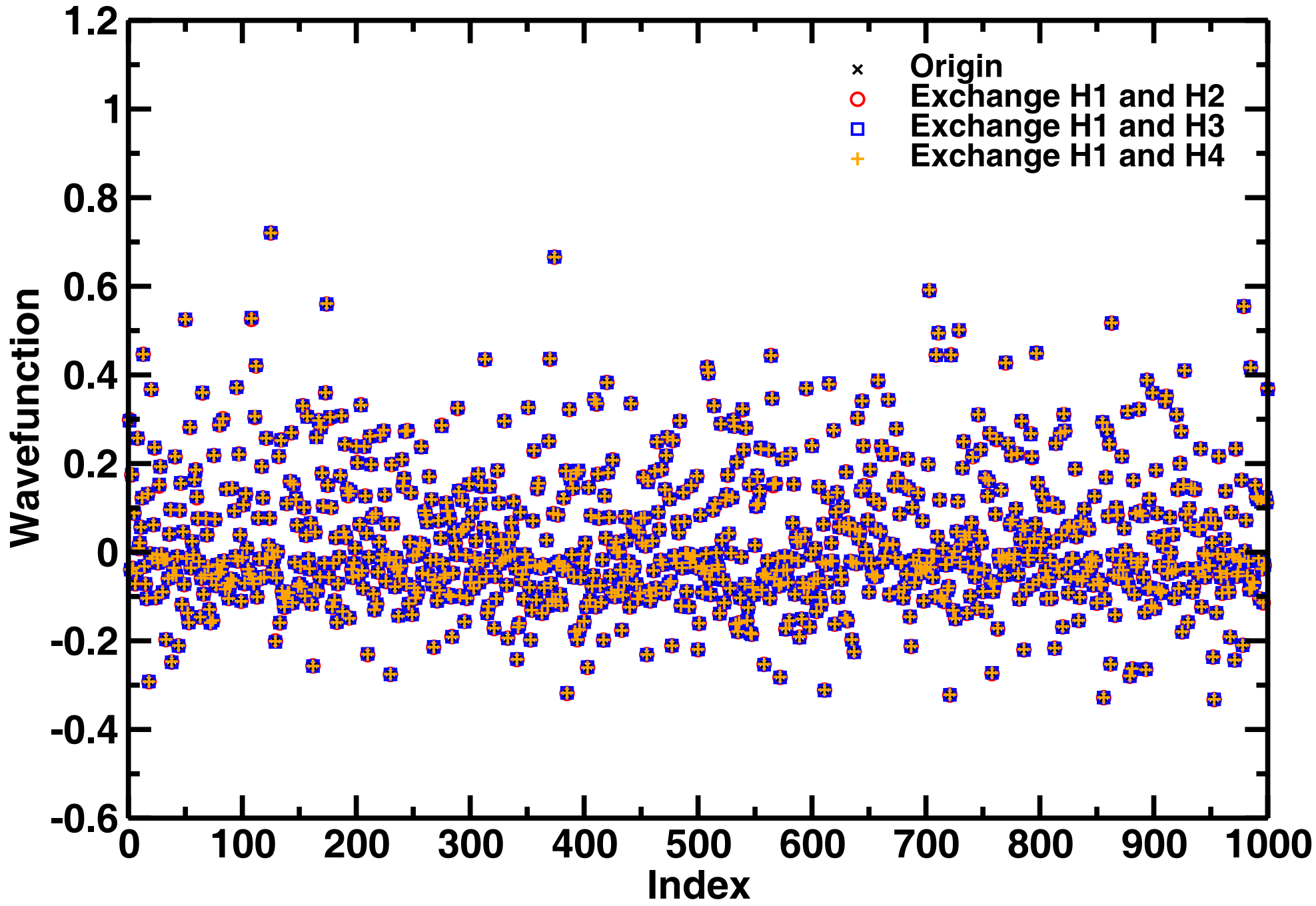

Suppl. Fig. 7. The first symmetric C-H stretching excited-state WFs values evaluated at 1000 MC-sampled configurations of the CH_4 molecule. All labels and legends are the same as in Suppl. Fig. 6. Source data are provided as a Source Data file.

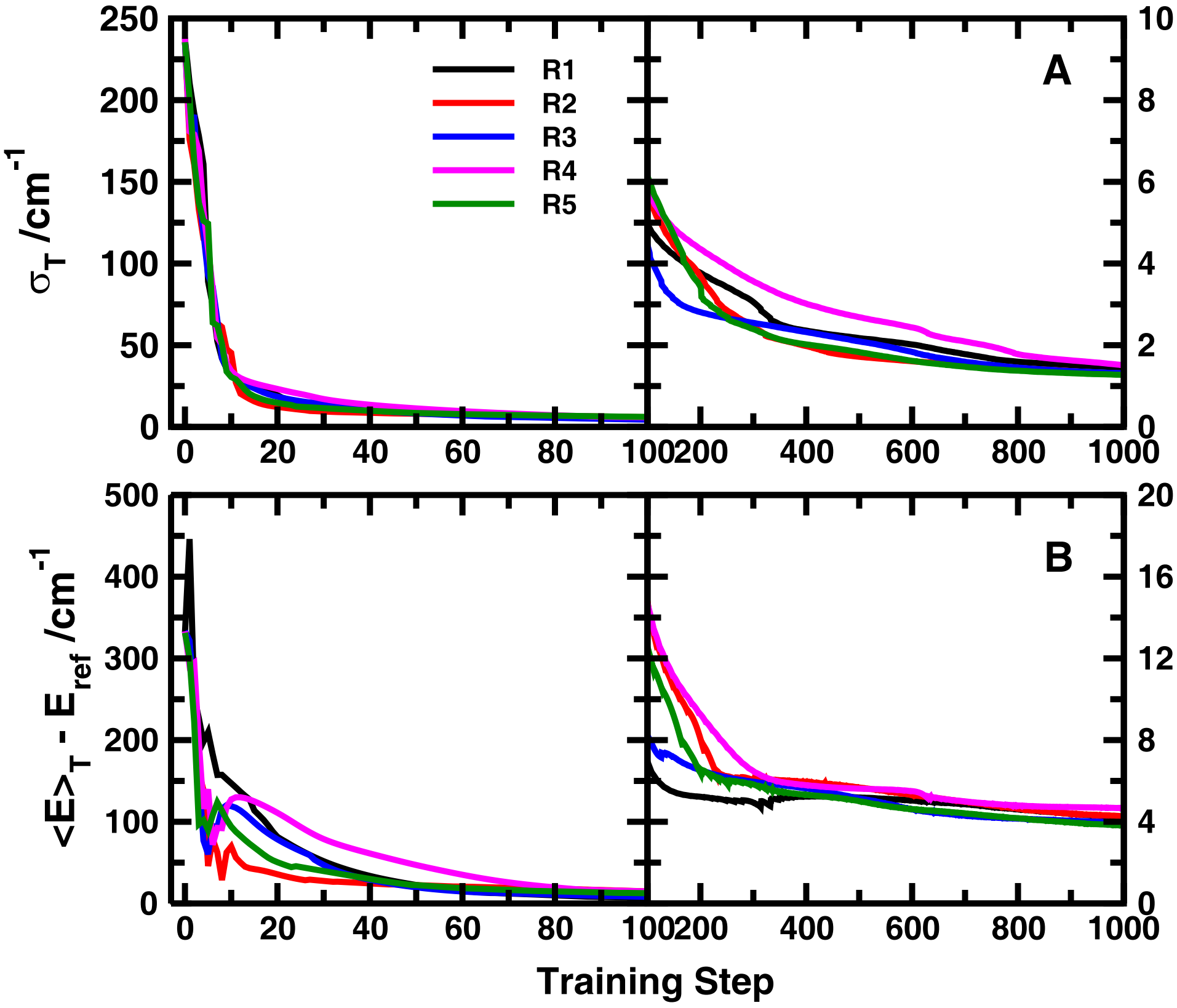

Suppl. Fig. 8. (A) The training-point variance σ_{T} and (B) the average energy $\langle E\rangle_{\mathrm{T}}$ relative to the DMC value of 22398.62±0.49 cm^{-1} for 5 replications, labeled R1–R5, as a function of training step for the ground state of C_3H_8 starting from $\psi^0(\vec{\boldsymbol{r}})$ obtained by fitting normal mode WF. Source data are provided as a Source Data file.

Supplementary references

1 Handy, N. C. The derivation of vibration-rotation kinetic energy operators, in internal coordinates. *Mol. Phys.* **61**, 207–223 (1987).

2 Manzhos, S. & Carrington, T. Using an internal coordinate Gaussian basis and a space-fixed Cartesian coordinate kinetic energy operator to compute a vibrational spectrum with rectangular collocation. *J. Chem. Phys.* **145**, 224110 (2016).

3 Anderson, J. B. Quantum chemistry by random walk. H ^2P, H^+_3 D_{3h} $^1A'_1$, H_2 $^3\Sigma^+_u$, H_4 $^1\Sigma^+_g$, Be ^1S. *J. Chem. Phys.* **65**, 4121–4127 (1976).

4 Suhm, M. A. & Watts, R. O. Quantum Monte Carlo studies of vibrational states in molecules and clusters. *Physics Reports* **204**, 293–329 (1991).

5 Kosztin, I., Faber, B. & Schulten, K. Introduction to the diffusion Monte Carlo method. *Am. J. Phys.* **64**, 633–644 (1996).

6 Behler, J. Neural network potential-energy surfaces in chemistry: a tool for large-scale simulations. *Phys. Chem. Chem. Phys.* **13**, 17930–17955 (2011).

7 Chen, J., Xu, X., Xu, X. & Zhang, D. H. A global potential energy surface for the H_2 + OH ↔ H_2O + H reaction using neural networks. *J. Chem. Phys.* **138**, 154301 (2013).

8 Jiang, B. & Guo, H. Permutation invariant polynomial neural network approach to fitting potential energy surfaces. *J. Chem. Phys.* **139**, 054112 (2013).

9 Jiang, B., Li, J. & Guo, H. Potential energy surfaces from high fidelity fitting ofab initiopoints: the permutation invariant polynomial - neural network approach. *Int. Rev. Phys. Chem.* **35**, 479–506 (2016).

10 Fu, B. & Zhang, D. H. Ab Initio Potential Energy Surfaces and Quantum Dynamics for Polyatomic Bimolecular Reactions. *J. Chem. Theory Comput.* **14**, 2289–2303 (2018).

11 Behler, J. Four Generations of High-Dimensional Neural Network Potentials. *Chem. Rev.* **121**, 10037–10072 (2021).

12 Manzhos, S. & Carrington, T., Jr. Neural Network Potential Energy Surfaces for Small Molecules and Reactions. *Chem. Rev.* **121**, 10187–10217 (2021).

13 Fu, B. & Zhang, D. H. Accurate fundamental invariant-neural network representation of ab initio potential energy surfaces. *Natl Sci. Rev.* **10**, nwad321 (2023).

14 Wang, X. G. & Carrington, T., Jr. Using experimental data and a contracted basis Lanczos method to determine an accurate methane potential energy surface from a least squares optimization. *J. Chem. Phys.* **141**, 154106 (2014).

15 Zhao, Z. *et al.* Full-dimensional vibrational calculations of five-atom molecules using a combination of Radau and Jacobi coordinates: Applications to methane and fluoromethane. *J. Chem. Phys.* **144**, 204302 (2016).
