# Supplementary material for: Solving the vibrational Schrödinger equation with artificial neural networks
Source: Nat Commun. 2026 Jun 20;17:7789. doi: 10.1038/s41467-026-74537-4 (PMC13438559; doi:10.1038/s41467-026-74537-4)
Supplement: Supplementary file 2 [file 41467_2026_74537_MOESM2_ESM.pdf]

nature portfolio

Peer Review File

Solving the vibrational Schrödinger equation with artificial neural networks

Corresponding Author: Professor Dong Hui zhang

This file contains all reviewer reports in order by version, followed by all author rebuttals in order by version.

Attachments originally included by the reviewers as part of their assessment can be found at the end of this file.

Version 1:

Reviewer comments:

Reviewer #1

(Remarks to the Author)
The manuscript makes further and worthy progress on the way to the use of machine learning (ML), in particular neural networks, to compute vibrational energy levels and transitions of polyatomic molecules. Despite the fact that basis precepts therefore have been known for a couple decades, this research direction has made, unfortunately, surprisingly (considering the strides of ML elsewhere in computational chemistry and physics) little progress. This work presents further and important progress by computing a number (albeit a small number) of energy levels of methane and even C3H8 - bigger molecules than done before with ML methods. The use of KEO in space-fixed Cartesian coordinates addresses the issue of dealing with complex KEO in substance. This is the way to go and the authors took that way.
I therefore am fundamentally for the publication. There are also questions and comments that I would like to see addressed before publication. They are:

- why are there not all levels corresponding to 4 fundamental frequencies of methane? Please also add frequencies to Table 1 besides level energies.

- did the authors try harmonics and combination bands and what was the outcome of those attempts?

- I think the arguments about the need to compute integrals in Eq.1 and then a related argument after Eq. 5 is forced: Eq. 1 is essentially collocation and can work without converging the integrals (see the example of using non-integrable basis functions in Mathematics, 6, 253 (2018) that rubs in the point of the absence of the need to converge integrals). Either way (i.e. either in Eq. 1 or Eq. 5) the sum is over training points. It is precisely because it is collocation-like and does not have to converge integrals that arbitrary point sets can be used. I suggest therefore tuning related parts of the text.

- I would like to see more explanation of why this NN architecture was used (i.e. 3 hidden layers).

Reviewer #2

(Remarks to the Author)
see attached pdf-file

Reviewer #4

(Remarks to the Author)
This manuscript reports a potentially interesting study about the solution of the time-independent nuclear Schrödinger equation. The manuscript needs to be improved significantly before it gets published.

Since this is the first paper of perhaps a series of papers on the topic (the authors mention this at the end of their summary section), the notation the authors use must be as good as possible. This is definitely not yet the case. I request that the authors overview their notation and make the necessary (mostly trivial) changes. For example, Fit(N,n1,n2) is defined on p. 5, but there are four arguments of Fit() later, like Fit(16200,45,45,5). The same holds for Train(). N is not defined in Eqs. (4)-

(6), which is especially bad in the present case as I'm not sure that Eq. (6) is correct (in its present form). Later N is said to be the number of fitting points. Note also that M is not defined in Eq. (1). There are many more minor issues that authors must correct (e.g., do not capitalize 'where' after the equations).

I do not see how the authors enforce translational, rotational, and permutational symmetry if they indeed use a kinetic energy operator in Cartesian coordinates (the discussion around Eq. (6) is not clear to me, especially in relation to the neglect of rotations). Perhaps this should be clearly discussed in the revised manuscript.

I suggest that the authors discuss the orthogonality of the vibrational wavefunctions, part of the usual solution of the Schrödinger equation. In fact in many applications the wavefunctions are even more important than the vibrational eigenenergies. How strictly are the vibrational wavefunctions orthogonal to each other? Can they be used to compute, for example, expectation values? This may need to be discussed in the revised manuscript.

At the moment the authors also do not discuss the symmetry of the vibrational wavefunctions (and their numerical stability with respect to the symmetry). This should also be remedied.

Is there a reason that the authors do not report the much larger number of combination and overtone states in this manuscript? Can they get the correct ordering of nearby states? How can they make sure that they are not missing any states from their computation(s)?

I'd like to read a couple of sentences about the inclusion of rotation in the nuclear motion Hamiltonian. In many applications the knowledge of rotational-vibrational eigenstates is certainly more important than that of vibrational states. The inclusion of the rotation would increase state density tremendously (especially for larger molecules). That would require increased precision from the calculations. Anything important the authors think about this should be included in the revised manuscript.

How do the authors see the cases of fluxional and quasistructural molecules? What if the normal modes are not good approximations of the true vibrational wave functions? How about torsional states corresponding to coupled large-amplitude motions?

The accuracy of the vibrational eigenstates depends really on the accuracy of the PES. Thus, comparing the computed eigenenergies to experiment is not really useful (see the Abstract), the calculated values (and the wave functions) should be compared to those obtained by a variational approach using an exact kinetic energy operator and exactly the same PES and masses.

In Table I, please subtract the ZPVE from the computed energy values, just like done in Table II.

Fix the relatively large number of "language" issues, e.g., 'expansive' -> expensive, quadrature pointS, equations cannot be converted to a 'linear algebra problem', state that you are solving the time-independent Schrödinger equation, correct 'great progresses', 'augment an addition term', 'relative large', 'training date set', and 'learning rate becomes small', it is unclear why you need to 'evaluate' a kinetic energy operator.

The title is missing from Ref. 41.

Version 2:

Reviewer comments:

Reviewer #1

(Remarks to the Author)
The authors responses are reasonable, and I can recommend acceptance.

(Remarks on code availability)

Reviewer #4

(Remarks to the Author)
I appreciate the time and effort the authors have invested in revising their manuscript. While the revised version represents a significant improvement over the original, I still disagree with some of the statements. For example, (a) there are multiple codes widely used codes based on a numerical representation of the exact kinetic energy operator in internal coordinates, so the phrase “increasingly efficient kinetic energy operators” is simply incorrect, (b) approaches based on the Smolyak grid can be applied to systems with substantially more than six atoms, and (c) VCI calculations based on the Eckart-Watson Hamiltonian have been done on selected states of larger than 6-atomic systems.

In addition, the authors should be mindful of several important limitations of their approach (besides the generation of an accurate PES). First, I have doubts that the authors will be able to use their technique to compute a large number of vibrational states of fluxional molecules, but this should not prevent the present contribution from publication. Second, unless they compute thousands —if not hundreds of thousands— of vibrational eigenvalues (as regularly done for smaller

systems these days), the practical value of their method for most experimentalists will remain rather limited. Third, for strongly bound oligomers, such as water clusters, the main challenge lies in describing highly excited intermolecular modes, while the intramonomer vibrations correspond to resonances rather than bound states. Fourth, for larger systems, the inclusion of rotational degrees of freedom is essential. I hope these issues will be addressed in the authors' future work.

(Remarks on code availability)

Open Access This Peer Review File is licensed under a Creative Commons Attribution 4.0 International License, which permits use, sharing, adaptation, distribution and reproduction in any medium or format, as long as you give appropriate credit to the original author(s) and the source, provide a link to the Creative Commons license, and indicate if changes were made.
In cases where reviewers are anonymous, credit should be given to 'Anonymous Referee' and the source.
The images or other third party material in this Peer Review File are included in the article's Creative Commons license, unless indicated otherwise in a credit line to the material. If material is not included in the article's Creative Commons license and your intended use is not permitted by statutory regulation or exceeds the permitted use, you will need to obtain permission directly from the copyright holder.
To view a copy of this license, visit https://creativecommons.org/licenses/by/4.0/

Response to Reviewers

Solving the vibrational Schrödinger equation with artificial neural networks

Shuaishuai Zhao & Dong H. Zhang*

Corresponding author: zhangdh@dicp.ac.cn

We would like to thank the three reviewers for the insightful comments which are very helpful for the improvement of our manuscript. We have carefully read those comments and replied to them point-by-point as follows. The revisions are indicated by blue fonts in the manuscript.

1. Response to the comments of Reviewer #1

Reviewer #1 (Remarks to the Author):

The manuscript makes further and worthy progress on the way to the use of machine learning (ML), in particular neural networks, to compute vibrational energy levels and transitions of polyatomic molecules. Despite the fact that basis precepts therefore have been known for a couple decades, this research direction has made, unfortunately, surprisingly (considering the strides of ML elsewhere in computational chemistry and physics) little progress. This work presents further and important progress by computing a number (albeit a small number) of energy levels of methane and even C3H8 - bigger molecules than done before with ML methods. The use of KEO in space-fixed Cartesian coordinates addresses the issue of dealing with complex KEO in substance. This is the way to go and the authors took that way.

I therefore am fundamentally for the publication. There are also questions and comments that I would like to see addressed before publication. They are:

- why are there not all levels corresponding to 4 fundamental frequencies of methane? Please also add frequencies to Table 1 besides level energies.

We thank the reviewer for the question. We have added the fourth fundamental frequency of methane in Table I in term of both frequencies and level energies.

- did the authors try harmonics and combination bands and what was the outcome of those attempts?

We thank the reviewer for the question, and we presume the "harmonics" here should be "overtone". We have calculated the energy levels for the overtone (0200) state and the combined (1100) state, and included them in Table I as well. We revised the 4th paragraph on Page 11 as follows: *In Table I, we show* $\langle E \rangle_R$ *and* σ_R *for the 7 selected vibrational states of the* CH_4 *molecule, including the ground state, four fundamental excited states, one overtone (0200) state, and one combined (1100) state, in terms of both frequency and level energy. Those results were obtained from 5 replications by training* $\psi^1(\vec{r})$ *with 500 training steps, using 2801 (10,45,45,5,1) parameters for the four fundamental excited states, and 4631 (10,60,60,5,1) parameters for the overtone (0200) and combined (1100) states. The difference between* $\langle E \rangle_R$ *and the reference value is less than 0.1* cm^{-1} *except for the symmetric stretch (1000) state of 0.42* cm^{-1} *and the overtone (0200) state of -0.41* cm^{-1}*.*

- I think the arguments about the need to compute integrals in Eq.1 and then a related argument after Eq. 5 is forced: Eq. 1 is essentially collocation and can work without converging the integrals (see the example of using non-integrable basis functions in Mathematics, 6, 253 (2018) that rubs in the point of the absence of the need to converge integrals). Either way (i.e. either in Eq. 1 or Eq. 5) the sum is over training points. It is precisely because it is collocation-like and does not have to converge integrals that arbitrary point sets can be used. I suggest therefore tuning related parts of the text.

We thank the reviewer for this valuable comment. We fully agree with the reviewer that the selection of arbitrary point sets for training is similar in spirit to that of the collocation method. However, in Eq. (4) and (5), we introduced a weight for each point which is absent in collocation method. This weight function plays an important role in our training process as shown in result section. Therefore, we feel it is reasonable to retain Eqs. (3)-(5) in our manuscript. While Eq. (1) and (2) were used in several preceding neural network studies, so we refer to them as the common way. We employed these two equations to calculate sampling variance and energy later. Thus, we believe it is worthwhile to keep them for clarity. It is worth noting that Eq. (3)-(5) are independent of Eq. (1)-(2), and all were used in our calculations. Finally, to highlight the similarity between the arbitrary point set used in this study and in the

collocation method, we have added the following paragraph on Page 5: *It is worth noting that the selection of arbitrary point sets in training is similar in spirit to that of the collocation method, which was introduced by Yang and Peet into chemical community using the same number of basis functions and collocation points. Later, Manzhos and Carrington proposed the rectangular collocation method by using more collocation points than the basis function. These collocation points can be randomly sampled in configuration space where the wave functions are nonzero to achieve higher efficiency in calculations.*

- I would like to see more explanation of why this NN architecture was used (i.e. 3 hidden layers).

We thank the reviewer for the suggestion. We have carefully tested various neural network architectures, and found a NN with three hidden layers offers the best overall performance, in terms of accuracy and efficiency. Accordingly, we have added the following sentence on Page 8: *We have carefully tested various neural network architectures, and found an NN with three hidden layers offers the best overall performance in terms of accuracy and computational efficiency for both the fitting and the subsequent training processes.*

2. Response to the comments of Reviewer #2

Reviewer #2 (Remarks to the Author):

In this paper, the authors present an artificial neural network implementation for solving the Schrödinger equation for the vibrational states of small molecular systems. They focus on the molecules methane (CH4) and propane (C3H8). They demonstrate that the ground state energy can be obtained with an accuracy of 1 cm^{-1}, which is comparable to the reference state determined using diffusion Monte Carlo calculations. Similarly, they show that vibrational energies for selected modes (the three C-C-C stretching and bending excitations) can be obtained within experimental uncertainty limits.

While the theoretical treatment of vibrational states is important due to the significance of vibrational spectroscopy in science and technology, the manuscript cannot be considered for publication for the reasons outlined below. As the entire premise of the article is based on erroneous methodological principles, it cannot be improved through revision. For this reason, rejection of the article is recommended.

1) Conceptual Errors: One of the biggest misconceptions when solving Schrödinger's equation is the idea that energy is the main focus of the calculation. This is, of course, not true: energy is a control variable, and the true target is the wave function (WF). According to Erwin Schrödinger's initial rationale, every property of the target system is implicitly described by the wave function and can be accessed by applying the relevant operator.

For this reason, wave functions must adhere to certain mathematical principles, foremost of which are normalization and orthogonality between different states. On pages 3 and 4, the authors explicitly remark:

> "*By initializing a NN with the normal mode WF for an excited state of a molecule, we can obtain accurate energy and WF for the state without imposing any orthogonality condition. We demonstrate our new method on the CH4 molecule, with spectroscopically accurate energies for ground and several excited states reported* "

This key statement lies at the heart of the methodology described and is directly related to the misconception regarding the solution to Schrödinger's equation mentioned above. While, in the context of vibrational spectroscopy, the energy eigenvalues of the individual states can be directly linked to the wave number of the absorbed photons, analysis of the wave functions (particularly the transition dipole moments and polarizabilities) is required to calculate infrared intensities and Raman activities.

Thus, avoiding a rigorous quantum mechanical treatment by sacrificing proper orthogonality may result in an accelerated framework to access wave numbers, but any calculation of vibrational transitions will ultimately appear unfeasible or – in the best case – be highly inaccurate. Although a very robust framework was established decades ago for the prediction of fundamental transitions (i.e. from the ground state to the first excited state), most modern research into the calculation of vibrational properties focuses on higher-order transitions relevant for IR and Raman spectroscopy in near-infrared regime. The latter are fundamentally more complex, but also provide significantly more data on chemical systems, as overtone and combination bands are accessible. A theoretical treatment of this highly complex phenomena requires access to the associated intensities / activities.

From the organisation of the manuscript, it seems that the authors are unaware of this issue, or are ignoring its implications. Due to the proposed methodology's inability to provide properly orthogonalised wave functions, which is inherent in the design of the

neural network, the article must be considered as a proof of concept for a rather questionable methodology. A number of more suitable approaches are currently emerging in the contributions by different research groups (e.g. Refs 15 to 26 in this work).

We thank the reviewer for pointing out the importance of orthogonality between wave functions. In fact, it is straightforward to prove that the eigenfunctions for non-degenerate states are orthogonal for the molecular Hamiltonian operator which is a Hermitian operator as follows:

$$\langle \varphi_i | \hat{H}\, \varphi_j \rangle = E_j \langle \varphi_i | \, \varphi_j \rangle = \langle \hat{H} \varphi_i | \, \varphi_j \rangle = E_i \langle \varphi_i | \, \varphi_j \rangle,$$

or

$$(E_i - E_j) \langle \varphi_i | \, \varphi_j \rangle = 0$$

where φ_i and φ_j are eigenfunctions for $\hat{H}$ with eigenvalue E_i and E_j, respectively. Since the state are non-degenerate, we have $E_i \neq E_j$, consequently $\langle \varphi_i | \, \varphi_j \rangle = 0$.

Therefore, it is not necessary to enforce mutual orthogonality between wave functions in a calculation as long as one can obtain well converged wavefunctions.

To verify the statement above, we show in Table S3 the overlap matrix for all the CH_4 vibrational states with energies shown in Table I. As can be seen, these states are indeed orthogonal within numerical uncertainty. We hope the reviewer has no further questions about this issue.

To highlight the importance of orthogonality between wave functions, we added the following sentence on Page 4 "*Because the Hamiltonian operator for a molecular system is a Hermitian operator, the calculated WFs are mutual orthogonal for non-degenerate eigenstates within numerical uncertainty, provided they are sufficiently accurate*.", and the following paragraph on Page 12 "*Table S3 shows the overlap matrix for all the CH_4 vibrational states that are listed in Table I. The diagonal elements of this matrix indicate that these WFs are normalized. Most off-diagonal elements are in the order of 10^{-4}, some of them are even smaller than 10^{-4}. The largest overlap element is -8.4×10^{-3} for the (1000) and (0200) states. Therefore, all the calculated WFs for the vibrational states are indeed orthogonal within numerical uncertainty.*"

2) Inadequate Example Systems: In the article, the authors discuss results for individual states of two simple molecules being CH4 and C3H8. By contrast, the vast majority of authors in this field compare their results to reference data of well-established benchmark sets, primarily the VIBFREQ1295 [1] and V30 [2] benchmarks, or the work of Barone et al. [3]. The authors should have considered calculating data for a few representative examples, or at least comparing their data to the published values for CH_4.

We thank the reviewer for the suggestion. Since this manuscript primarily presents a new methodology for calculating vibrational eigenstates using NN, we selected the CH_4 molecule to illustrate the new method in detail because fully converged benchmark results are available for it using the variation method on the potential energy surface employed here. The C_3H_8 molecule was chosen to demonstrate the capability of the method for larger systems. To the best of our knowledge, C_3H_8 molecule is the largest molecular system for which accurate (~1 cm^{-1}) vibrational state calculations on a given potential energy surface have been reported, as indicated by the excellent agreement with DMC benchmarks. Therefore, we think it is appropriate to use CH_4 and C_3H_8 molecules to introduce and validate the new method.

We plan to carry out further calculations for additional systems, such as those provided in VIBFREQ1295, V30, and Barone et al., in follow-up studies. Because our approach requires a global potential energy surface for each system, we will need to construct high-quality PESs if they are not available, which means that these calculations cannot be finished in a short period of time.

Finally, we note that the CH_4 values we compare our results to are published data from Ref. [40] in the original manuscript, now in Ref. [63].

In response to the reviewer's comment, we have added the following sentences at the last paragraph of the manuscript: *We will carry out further calculations for both semirigid and fluxional systems, such as those provided in VIBFREQ1295, V30, and in the work of Barone et al., in follow-up studies. Because our approach requires a global potential energy surface for each system, we will need to construct high-quality PESs if they are not available.*

As such, the presentation of individual excitation appears arbitrary. Similarly, comparison to experiment is in many cases misleading ("correct result for wrong reason") and the authors are highly encouraged to adhere to the "best practice" standards of the vibrational spectroscopy community.

We apologize for the apparent arbitrariness in the presentation of individual

excitations. As the primary aim of this manuscript is to introduce and validate the new method and we need to calculate these excited states one by one, we initially only presented a few excitations for CH_4 and C_3H_8. In revision, we have addressed this by adding the fourth fundamental excitation, one overtone, and one combined state for CH_4 to provide a more comprehensive demonstration.

We fully agree with the reviewer on the comparison to experiment. For CH_4, we have compared the calculated vibrational energies with those from a variational approach that uses an exact kinetic energy operator, the same PES, and the same masses. Furthermore, in revision, we have added a comparison of the expectation values. The excellent agreement for both vibrational energies and expectation values, as shown in Table I and Tables S4-7, not only demonstrates the accuracy of our NN method but also confirms the correctness of the kinetic energy operator given in Eq. (6).

However, for C_3H_8 - a molecule with 11 atoms - the eigenvalue for the ground state, (i.e. the zero point energy) obtained by using DMC is, to our knowledge, the only theoretical value accurate to within ~cm^{-1} on a given PES. As shown in Table II, the agreement between the DMC benchmark and our result is perfect.

Furthermore, the PES used for C_3H_8 in this study is a high-quality PES fitted using the fundamental invariant neural network (FI-NN) method, based on more than 100,000 ab initio energies calculated on CCSD(T)-F12a/aug-cc-pvtz level of theory. Therefore, in the absence of accurate theoretical results for excited states, we believe it is meaningful to compare our calculated excitation energies with experimental values. The good agreement not only demonstrates the accuracy of our method for molecules with more than 10 atoms but also verifies the accuracy of the PES used in the calculation.

<u>3) Ignoring/Misciting literature:</u> On page 2, the authors provide a bulk citation of previous works:

> "*In the past decades, neural networks have also been used to solve vibrational SE, however, mostly for synthetic systems such as one or multi-dimensional harmonic oscillators (15-26).*"

This statement is imprecise and does not do justice to the contributions made to these works. For instance:

– Lagaris et al. (Ref. 16) included results obtained for a three-dimensional system of coupled anharmonic oscillators, which is comparable to the treatment of a non-linear three-atomic system.

– Nakanishi and Sugawara (Ref. 17) were considering a 1D double-well potential linked to quantum tunneling in addition to the harmonic oscillator.

– Mills and coworkers (Ref. 20) demonstrated their methods including 2D double-well inverted Gaussian potentials as well as entirely random 2D potential grids.

– Hong et al. (Ref. 23) applied their functional tensor network to a three-body coupled oscillator, thus giving rise to correlation and entanglement.

– Gamper et al. (Ref. 24) applied a PINN to 1D anharmonic oscillators, rigid rotors, periodic finite potentials as well as the hydrogen molecule and the torsional tunneling of the phenole molecule.

– Domingo et al. (Ref 25) considered 1D anharmonic and polynomial potentials.

Underselling the contributions of other research groups in this way is certainly not adequate and is unacceptable in any scientific article, regardless of the journal in question.

We thank the reviewer for the comments. We agree with the reviewer that the sentence, i.e. *In the past decades, neural networks have also been used to solve vibrational SE, however, mostly for synthetic systems such as one or multi-dimensional harmonic oscillators (15-26),* is not sufficient to describe the contributions from the proceeding researches in this area, but we don't have intention to undersell these contributions.

We added the following sentences on Page 2-3: *In the past decades, neural networks have also been used to solve vibrational SE. Lagaris et al. studied a three-dimensional system of coupled anharmonic oscillators, which is comparable to the treatment of a non-linear three-atomic system (Ref. 16). Nakanishi and Sugawara considered a 1D double-well potential linked to quantum tunneling in addition to the harmonic oscillator (Ref. 17). Mills and coworkers demonstrated their methods including 2D double-well inverted Gaussian potentials as well as entirely random 2D potential grids (Ref. 20). Hong et al. applied their functional tensor network to a three-body coupled oscillator, thus giving rise to correlation and entanglement (Ref. 23). Gamper et al. applied a PINN to 1D anharmonic oscillators, rigid rotors, periodic finite potentials as well as the hydrogen molecule and the torsional tunneling of the phenol molecule (Ref. 24). Domingo et al. considered 1D anharmonic and polynomial potentials (Ref 25).*

In addition to these oversights, a plethora of literature exists on accurate, grid-based methods focused on the precise calculations of Schrödinger's equation, including

many contributions to problems associated to molecular vibrations. This fundamental research area is not mentioned at all in the manuscript.

We thank the reviewer for the comments. We have added a paragraph on Page 2 that briefly summarizes the key advances in accurate, grid-based methods for the precise calculations of the Schrödinger's equation, along with 14 reference papers as follows: *Over the past decades, this rigorous quantum method has been evolving substantially mainly through the development of increasingly efficient basis functions and kinetic energy operators[1-14]. It is now feasible to perform fully variational computations for various small molecules containing up to six atoms in full dimensions. However, due to the exponential scaling of this kind of calculation, it is not feasible to extend the method to systems containing far more than six atoms in the foreseeable future.*

<u>4) Wrong use of scientific terms:</u> I was surprised to read the term 'asymmetric stretch' in a contribution submitted to a Nature journal. The correct term is 'antisymmetric stretch', since these contributions arise from an antisymmetric superposition of the isolated stretch modes of the associated bonds (i.e. opposite symmetry). In contrast, 'asymmetric' implies a complete absence of any kind symmetry.

The authors will certainly argue that it is acceptable to use an incorrect scientific term because it is commonly used by the majority of researchers in the field of chemistry. I sharply disagree here.When considering contributions by leading researchers in the field, such as Yukihiro Ozaki [4], Guntram Rauthut [5] and Vincenzo Barone [6], one consistently encounters the correct term 'antisymmetric'. There is even a dedicated contribution by a former associate editor of the journal Applied Spectroscopy entitled 'Molecular vibrations are not asymmetric' [7], discussing the origins of the correct term at great length. Also companies such as Bruker are committed to establishing a correct terminology, as can be seen from their online tutorial, 'Guide to Infrared Spectroscopy' [8].

Given that the anti-symmetric superposition arises directly from degeneracy in the quantum mechanical description of oscillator wave functions associated with the isolated stretch vibrations, the use of the wrong terminology in a scientific contribution focused on a novel treatment of Schrödinger equation for vibrational problems is not without a certain irony.

We thank the reviewer for the comments. We apologize for the incorrect terminology and have changed all "asymmetric" to "antisymmetric" throughout the manuscript.

Taking into account this particular shortcoming in view of the conceptual errors highlighted above, it appears that the authors are not particularly familiar with the underlying theoretical foundations and the best practice adopted by leaders in the field. As a consequence, this manuscript is not acceptable for publication in any peer-reviewed scientific journal focused on chemical theory.

5) Missing Description of the Methodology: Although the authors explained their choices regarding the neural network in great detail, the main article does not provide a number of key pieces of information. These include:

– Details on the calculation of the potential energy surface. The authors mention the use of diffusion Monte Carlo, however, the applied software, the calculation settings or detailed reference data are not provided at all.

We thank the reviewer for the comments. The potential energy surfaces (PESs) used in this work are published surfaces; readers can find their details in the respective references (CH_4: Ref. [63]; C_3H_8: Ref. [65]).

Regarding the diffusion Monte Carlo (DMC) calculations, we used a simple in-house program rather than existing software. All parameters for the C_3H_8 DMC calculations are listed in the Supporting Information, and the program has been uploaded. And we have added the following sentence on Page 13: *See the SI for details of the DMC calculation.*

– No discussion regarding the actual implementation is provided in the main article. This renders statements for routines such as Fit(16200, 45, 45, 5) and Train(18600, 45, 45, 5) frequently encountered in the article inappropriate. While Fit() is briefly mentioned on page 5, it is defined as Fit(N, n1, n2), i.e. using three arguments and not four. Most likely, the authors forgot to include n0 in the definition. In contrast to Fit(), the routine Train() is not defined at all.

We thank the reviewer for the comments. We have defined the arguments for Fit on Page 6 and Train on Page 7.

Very little information is provided in the supplementary material, so a number of key settings are missing and must be discussed in the main article in order for readers to follow the methodology, which currently is not possible.

We thank the reviewer for pointing out this issue. To clarify the overall computational procedure, we have added a flowchart as Fig. S1. As shown in the figure, the following steps are required to solve the vibrational Schrödinger equation:

1. **Construct a normal mode wave function.** This is an easy initial step.
2. **Sample the wave function.** This is a routine sampling procedure using **the Monte Carlo (MC) method.**
3. **Initialize the parameters for the Gaussian function.** Detailed information is provided in the SI.
4. **Fit a wave function using a neural network (NN).** The NN architecture and the number of data points used for fitting are provided in the main text. Researchers familiar with NN fitting should be able to replicate this step.
5. **Train a wave function.** The loss functions are specified. In principle, one can write a program to perform the training of the neural network representing the wavefunction. However, if required, we are happy to share our training code.
6. **Calculate the sample-averaged energy, $\langle \boldsymbol{E} \rangle_{\boldsymbol{S}}$.** This involves using MC to generate a sufficient number of sampling points and calculating their energies using the kinetic energy operator given in Eq. (6) and potential energy surface.

Therefore, we believe that researchers familiar with neural networks can now follow this methodology to reproduce the results with a reasonable investment of time.

6) Minor points:

– On page 7 the authors note for the treatment of CH4:

> "*The input layer has 10 nodes, equal to the number of bond lengths in the molecule.*"

Most chemists will unanimously reject this statement, since there are only 4 bonds in the methane molecule. Most likely the authors also consider the H-H vectors as chemical bonds, which is not a correct way to describe the molecule, adding to the list of oversights and shortcomings regarding

chemical terminology.

We thank the reviewer for pointing out the problem. We have changed "bond lengths" into "internuclear distances" in the manuscript.

– Page numbers: The submission of the article with missing page numbers is a clear indication that the authors did not take the preparation of the article too seriously. This is inline with the many shortcomings discussed above and one can get the impression, that the entire project was considered as a quick contribution to ride on the current machine learning/AI hype. The missing quality in manuscript preparation just further underlines this point.

We thank the reviewer for pointing out the problem. We have added the page numbers in our revised manuscript.

– Level of English: While throughout large parts of the manuscript the level of English is adequate, there are still a few sentences with missing articles. Considering that we now live in the age of AI, an automated English check with any free AI agent should be a matter of minutes.

We thank the reviewer for the comments. We have done our best to correct the language problems, in particular the missing articles.

– References: Individual references (38, 41) are incomplete adding to the picture of a quickly prepared study. Also, considering the magnitude and importance of this work, a total reference count of 41 appears too small. The authors are encouraged to maximize the allowed number of references.

We thank the reviewer for the comments. We have fixed the problems with references (38, 41), and added a large number of new references relevant to this work.

In summary, this article presents an ill-conceived neural network implementation enabling the calculation of vibrational eigenenergies based on the Schrödinger equation. While being applicable to larger systems, the sacrifice of wave function orthogonality will greatly limit the framework for any useful prediction of molecular vibrational spectra in terms of intensities / activities. Added to this methodical short comings is the absence of any notable benchmarking, the use of wrong terminology, an inadequate summary of previous contributions to the field and missing details regarding the applied methodology.

From this perspective, the manuscript cannot be accepted for publication in any peer-reviewed scientific journal. Due to its many shortcomings, it cannot be improved through revision. For these reasons, rejection is the only possible recommendation.

References:

[1] "VIBFREQ1295: A New Database for Vibrational Frequency Calculation" J. C. Zapata Trujillo, L. K. McKemmish J. Phys. Chem. A 126 (2022) 4100 – 4122 DOI: 10.1021/acs.Jpca.2c01438

[2] "The V30 benchmark set for anharmonic vibrational frequencies of molecular dimers" J. Hoja, D. A. Boese J. Chem. Phys. 161 (2024) 234110 DOI: 10.1063/5.0238491

[3] "Accuracy Meets Interpretability for Computational Spectroscopy by Means of

Hybrid and Double-Hybrid Functionals” V. Barone, G. Ceselin, M. Fusè, N. Tasinato Front. Chem. 8 (2020) 584203 DOI: 10.3389/fchem.2020.584203

[4] “Two-Dimensional Correlation Analysis, Fourier Self-Deconvolution, and Second-Derivative Studies on Assignments of the CH2 Stretching Bands for Cast Films of 2-Dodecyl-, 2-Pentadecyl-, and 2-Octadecyl-7,7,8,8-Tetracyanoquinodimethane” H.-S. Wang, Y. Ozaki Appl. Spectrosc. 54 (2000) 692 – 698 DOI: 10.1366/0003702001949994

[5] “A combined variational and perturbational study on the vibrational spectrum of P2F4” G. Rauhut, T. Hrenar Chem. Phys. 346 (2008) 160 – 166 DOI: 10.1016/j.chemphys.2008.01.039

[6] “Interplay of Stereoelectronic and Vibrational Modulation Effects in Tuning the UPS Spectra of Unsaturated Hydrocarbon Cage Compounds” L. Paoloni, M. Fusè, A. Baiardi, V. Barone J. Chem. Theory Comput. 16 (2020) 5218 – 5226 DOI: 10.1021/acs.jctc.0c0064 5

[7] “Molecular Vibrations Are Not Asymmetric” J. A. de Haseth Appl. Spectrosc. 76 (2022) 150 –152 DOI: https://doi.org/10.1177/000370282110365

[8] “Guide to Infrared Spectroscopy” Bruker https://www.bruker.com/en/products-and-solutions/infrared-and-raman/ ft-ir-routine-spectrometer/ what-is-ft-ir-spectroscopy.html ; (02.08.2025) see attached pdf-file

3. Response to the comments of Reviewer #4

Reviewer #4 (Remarks to the Author):

This manuscript reports a potentially interesting study about the solution of the time-independent nuclear Schrödinger equation. The manuscript needs to be improved significantly before it gets published.

Since this is the first paper of perhaps a series of papers on the topic (the authors mention this at the end of their summary section), the notation the authors use must be as good as possible. This is definitely not yet the case. I request that the authors overview their notation and make the necessary (mostly trivial) changes. For example, Fit(N,n1,n2) is defined on p. 5, but there are four arguments of Fit() later, like Fit(16200,45,45,5). The same holds for Train(). N is not defined in Eqs. (4)-(6), which

is especially bad in the present case as I'm not sure that Eq. (6) is correct (in its present form). Later N is said to be the number of fitting points. Note also that M is not defined in Eq. (1). There are many more minor issues that authors must correct (e.g., do not capitalize 'where' after the equations).

We thank the reviewer for pointing out the notation and other minor problems. We have done our best to correct all these problems. Specifically, we have defined the arguments for Fit on Page 6 and Train on Page 7, N in Eq. (4)-(5), Na in Eq. (6), and M in Eq. (1). We also changed “Where” to “where” after the equations.

I do not see how the authors enforce translational, rotational, and permutational symmetry if they indeed use a kinetic energy operator in Cartesian coordinates (the discussion around Eq. (6) is not clear to me, especially in relation to the neglect of rotations). Perhaps this should be clearly discussed in the revised manuscript.

We thank the reviewer for the suggestion and have added a detailed derivation of the kinetic energy operator to the SI. As shown in the SI, the translational motion can be removed from the Hamiltonian rigorously. The remaining Hamiltonian contains terms that depend on the total angular momentum of the system J. In the present study, we only consider the case of J=0. Consequently, all the results presented in the manuscript pertain to the pure vibration of the molecules.

Permutational symmetry was not explicitly enforced in our calculations. In principle, permutationally symmetric wavefunctions could be represented using fundamental invariants neural networks (FI-NNs). However, this would substantially increase computational cost because the number of fundamental invariants is much larger than the number of internuclear distances, especially for larger molecules like C_3H_8. Representing antisymmetric wavefunctions (analogous to electronic wavefunctions) would be even more challenging.

Importantly, if the potential energy surface itself possesses permutational symmetry, numerically accurate solutions of the Schrödinger equation will naturally exhibit that symmetry, even without its explicit enforcement. A good example is the calculation of H_2O vibrational states using Jacobi coordinates, where the two hydrogen atoms are treated differently (e.g., one OH bond as $\vec{r}$, and the vector from its center of mass to the other H as $\vec{R}$). No permutational symmetry is enforced in this coordinate choice, yet the resulting wavefunctions correctly show the symmetry within numerical uncertainty.

To demonstrate that our obtained wavefunctions indeed exhibit the correct

permutational symmetry, we have added two figures (Figs. S4 and S5) showing one-dimensional cuts of the wavefunctions along each of the four C–H bonds in CH_4 for the ground and first symmetric C-H stretching excited state, respectively. As seen in these figures, all four cuts are identical for these two symmetric states. We have also added Figs. S6 and S7, which show the values of the wave functions at 1000 Monte Carlo sampled configurations before and after the permutation of two hydrogen atoms. Similarly, the wave functions are identical after permutation.

The following paragraph has been added to the manuscript (Page 12): *Figs. S4 and S5 show one-dimensional cuts of the wavefunctions along each of the four C–H bonds in* CH_4 *for the ground and first symmetric C-H stretching excited state, respectively. As seen in these figures, all four cuts are identical for these two symmetric states. We also show in Figs. S6 and S7 the values of the wave functions at 1000 Monte Carlo sampled configurations before and after the permutation of two hydrogen atoms. Similarly, the wave functions remain unchanged after permutation. Therefore, although the permutational symmetry was not enforced in our calculations, the obtained wavefunctions exhibit the proper symmetry.*

I suggest that the authors discuss the orthogonality of the vibrational wavefunctions, part of the usual solution of the Schrödinger equation. In fact in many applications the wavefunctions are even more important than the vibrational eigenenergies. How strictly are the vibrational wavefunctions orthogonal to each other? Can they be used to compute, for example, expectation values? This may need to be discussed in the revised manuscript.

We thank the reviewer for the important comments and suggestions. We pointed out in reply to Reviewer 2 that the wavefunctions for non-degenerated eigenstates are orthogonal for the Hamiltonian $\widehat{H}$, which is a Hermite operator. As can be seen from Table. S3, the 7 eigenfunctions with eigenvalues shown in Table I are indeed orthogonal within numerical uncertainties.

The calculated wave functions can be used to compute expectation values, as shown in Tables S4-7. We have added the following paragraph on Page 12: *To show further the obtained wave functions can be used to calculate accurate expectation values, we list the average values of the four CH bond lengths in Table S4 and their standard deviations in Table S5 for the ground and four fundamental excited states, in comparison with those from the variational method. First, one can see that the four average values of CH bond lengths and their standard deviations for all these states*

are both identical to the fourth decimal place, further verifying the permutation symmetry of the calculated wave functions. They are also identical to the variational results to the fourth decimal place, indicating the calculated wave functions using NN method are of high accuracy. In Tables S6 and S7, we show the average values and the standard deviations for six H-C-H angles for these five states. As for the CH bond lengths in Tables S4 and S5, all the average values and the standard deviations obtained from the NN wave functions agree perfectly with each other, and they also agree very well those from the variation calculation.

At the moment the authors also do not discuss the symmetry of the vibrational wavefunctions (and their numerical stability with respect to the symmetry). This should also be remedied.

We thank the reviewer for raising the point. As we understand it, the symmetry of the vibrational wavefunctions is closely related to the permutational symmetry. We have added some content on Page 12 and in SI regarding the permutational symmetry, and hope this addresses the symmetry issue properly.

Is there a reason that the authors do not report the much larger number of combination and overtone states in this manuscript? Can they get the correct ordering of nearby states? How can they make sure that they are not missing any states from their computation(s)?

We thank the reviewer for these questions. As shown in Table I, we are able to calculate the combination (1100) and the overtone (0200) states accurately for CH_4. However, since we need to calculate these levels one by one by starting from different initial approximated wave functions, it would be very expensive to report a large number of combination and overtone states. As also shown in Table I, we can get the correct ordering of nearby states, such as the (0010) and (0200) states, because we can converge the vibrational energy within 1 cm^{-1}.

The main aim of the present work is to develop a NN method to calculate accurate vibrational energies for molecules intractable to existing methods. While currently limited to a limited number of states, it yields accurate energies and wave functions for fundamental, low-energy combination, and overtone states in molecules exceeding ten atoms -- a capability unprecedented in the field.

I'd like to read a couple of sentences about the inclusion of rotation in the nuclear motion Hamiltonian. In many applications the knowledge of rotational-vibrational eigenstates is certainly more important than that of vibrational states. The inclusion of the rotation would increase state density tremendously (especially for larger molecules). That would require increased precision from the calculations. Anything important the authors think about this should be included in the revised manuscript.

We thank the reviewer for pointing out the rotational problem. We have added the last paragraph to address the issue: *Finally, we note that the Hamiltonian in Eq. (6) is for the total molecular angular momentum J=0, and all calculations presented in this work are for vibrational energy levels. In principle, one can use the rovibrational Hamiltonian in Eq. S3 to compute rovibrational energies. However, the inclusion of the rotation requires higher computational precision due to the small spacing of rotational energy levels, especially for larger molecules. Therefore, calculating accurate rovibrational levels is considerably more challenging and will require further effort.*

How do the authors see the cases of fluxional and quasistructural molecules? What if the normal modes are not good approximations of the true vibrational wave functions? How about torsional states corresponding to coupled large-amplitude motions?

We thank the reviewer for these important questions. As we mentioned in the discussion at the last paragraph of our manuscript, we have successfully applied the method to $(H_2O)_2$, $(H_2O)_3$, and very recently even to $(H_2O)_4$. We are able to calculate the ground state, almost all the intramolecular fundamental energy levels and some of the intermolecular energy levels, with high accuracy. Therefore, this NN based method can be used to study fluxional and quasi-structural molecules, as well as torsional states corresponding to coupled large-amplitude motions.

While, when the normal modes are not good approximations for the true vibrational wave functions as for intermolecular energy levels of water clusters, it is much hard to design appropriate initial wavefunctions to converge our calculation to the desired states. We have made some progress on this issue, and the results will be reported in a follow-up paper soon. However, considerable effort is still required to find a systematic way to tackle it.

The accuracy of the vibrational eigenstates depends really on the accuracy of the PES. Thus, comparing the computed eigenenergies to experiment is not really useful (see the Abstract), the calculated values (and the wave functions) should be compared to those obtained by a variational approach using an exact kinetic energy operator and

exactly the same PES and masses.

We fully agree with the reviewer and Reviewer 2 on the comparison to experiment. For CH_4, we have compared the calculated vibrational energies with those from a variational approach that uses an exact kinetic energy operator, the same PES, and the same masses. Furthermore, in revision, we have added a comparison of the expectation values. The excellent agreement for both vibrational energies and expectation values, as shown in Table I and Tables S4-7, not only demonstrates the accuracy of our NN method but also confirms the correctness of the kinetic energy operator given in Eq. (6).

However, for C_3H_8 - a molecule with 11 atoms - the eigenvalue for the ground state, (i.e. the zero point energy) obtained by using DMC is, to our knowledge, the only theoretical value accurate to within ~cm^{-1} on a given PES. As shown in Table II, the agreement between the DMC benchmark and our result is perfect.

Furthermore, the PES used for C_3H_8 in this study is a high-quality PES fitted using the fundamental invariant neural network (FI-NN) method, based on more than 100,000 ab initio energies calculated on CCSD(T)-F12a/aug-cc-pvtz level of theory. Therefore, in the absence of accurate theoretical results for excited states, we believe it is meaningful to compare our calculated excitation energies with experimental values.

In Table I, please subtract the ZPVE from the computed energy values, just like done in Table II.

In revised manuscript, we show vibrational energies for CH_4 in Table I in terms of both frequency and level energy.

Fix the relatively large number of "language" issues, e.g., 'expansive' -> expensive, quadrature pointS, equations cannot be converted to a 'linear algebra problem', state that you are solving the time-independent Schrödinger equation, correct 'great progresses', 'augment an addition term', 'relative large', 'training date set', and 'learning rate becomes small', it is unclear why you need to 'evaluate' a kinetic energy operator.

We have done our best to fix the “language” issue.

The title is missing from Ref. 41.

We added the title for Ref. 41.

Response to Reviewers

Solving the vibrational Schrödinger equation with artificial neural networks

Shuaishuai Zhao & Dong H. Zhang*

Corresponding author: zhangdh@dicp.ac.cn

We greatly appreciate the reviewers' time and effort in evaluating our revised manuscript. Those comments have helped us further improve the manuscript. We have carefully considered all the comments and responded to them point-by-point as follows.

1. Response to the comments of Reviewer #4

Reviewer #4 (Remarks to the Author):

I appreciate the time and effort the authors have invested in revising their manuscript. While the revised version represents a significant improvement over the original, I still disagree with some of the statements. For example, (a) there are multiple codes widely used codes based on a numerical representation of the exact kinetic energy operator in internal coordinates, so the phrase "increasingly efficient kinetic energy operators" is simply incorrect,

We thank the reviewer for the comment. We agree with the reviewer that the phrase "increasingly efficient kinetic energy operators" is incorrect. We have revised the corresponding sentence on Page 2: *Over the past decades, this rigorous quantum approach has been evolving substantially through the development of increasingly efficient basis functions and methods for evaluating kinetic energy operators.*

(b) approaches based on the Smolyak grid can be applied to systems with substantially more than six atoms, and (c) VCI calculations based on the Eckart-Watson Hamiltonian have been done on selected states of larger than 6-atomic systems.

We thank the reviewer for this comment. We agree with the reviewer's statements regarding approaches based on the Smolyak grid and VCI calculations based on the Eckart-Watson Hamiltonian. We added the following sentences on Page 2: *Approaches based on the Smolyak sparse-grid scheme have been developed and successfully applied to full-dimensional vibrational calculations on malonaldehyde, a molecule with nine atoms. Beyond these fully variational full-dimensional calculations, many approximate methods, most originally developed for electronic structure calculations, have been applied to compute vibrational energy levels of*

medium- to large-sized molecular systems with respectable accuracy. These methods are typically formulated within a Watson-type Hamiltonian framework and employ truncated representations of the potential energy surface, such as n-mode or Taylor expansions around the equilibrium geometry, while incorporating rotation-vibration interactions through the kinetic-energy operator.

In addition, the authors should be mindful of several important limitations of their approach (besides the generation of an accurate PES). First, I have doubts that the authors will be able to use their technique to compute a large number of vibrational states of fluxional molecules, but this should not prevent the present contribution from publication. Second, unless they compute thousands — if not hundreds of thousands — of vibrational eigenvalues (as regularly done for smaller systems these days), the practical value of their method for most experimentalists will remain rather limited. Third, for strongly bound oligomers, such as water clusters, the main challenge lies in describing highly excited intermolecular modes, while the intramonomer vibrations correspond to resonances rather than bound states. Fourth, for larger systems, the inclusion of rotational degrees of freedom is essential. I hope these issues will be addressed in the authors' future work.

We thank the reviewer for pointing out these important limitations of the proposed method. We agree that computing a large number of vibrational states for fluxional molecules, treating highly excited intermolecular modes and the resonance of intramonomer vibrations in strongly bound oligomers, and incorporating rotational degrees of freedom for larger systems remain important challenges.

We have been working to address these issues and made some progress. As noted in our previous response, we have successfully applied the method to calculate the zero point energy and the intramolecular fundamental energy levels of water clusters, including $(H_2O)_2$, $(H_2O)_3$, and $(H_2O)_4$. These results will be reported in follow-up papers. However, computing thousands of vibrational eigenvalues is expensive with the current implementation, because we need to calculate the eigenvalues one by one. We will address these remaining issues in future work.

Report on manuscript NCOMMS-24-29141A-Z entitled “Solving the vibrational Schrödinger equation with artificial neural networks“ by S. Zhao and D. H. Zhang

In this paper, the authors present an artificial neural network implementation for solving the Schrödinger equation for the vibrational states of small molecular systems. They focus on the molecules methane (CH_4) and propane (C_3H_8). They demonstrate that the ground state energy can be obtained with an accuracy of 1 cm^{-1}, which is comparable to the reference state determined using diffusion Monte Carlo calculations. Similarly, they show that vibrational energies for selected modes (the three C-C-C stretching and bending excitations) can be obtained within experimental uncertainty limits.

While the theoretical treatment of vibrational states is important due to the significance of vibrational spectroscopy in science and technology, the manuscript cannot be considered for publication for the reasons outlined below. As the entire premise of the article is based on erroneous methodological principles, it cannot be improved through revision. For this reason, rejection of the article is recommended.

<u>1) Conceptual Errors:</u> One of the biggest misconceptions when solving Schrödinger's equation is the idea that energy is the main focus of the calculation. This is, of course, not true: energy is a control variable, and the true target is the wave function (WF). According to Erwin Schrödinger's initial rationale, every property of the target system is implicitly described by the wave function and can be accessed by applying the relevant operator.

For this reason, wave functions must adhere to certain mathematical principles, foremost of which are normalization and orthogonality between different states. On pages 3 and 4, the authors explicitly remark:

> *“By initializing a NN with the normal mode WF for an excited state of a molecule, we can obtain accurate energy and WF for the state without imposing any orthogonality condition. We demonstrate our new method on the CH4 molecule, with spectroscopically accurate energies for ground and several excited states reported“*

This key statement lies at the heart of the methodology described and is directly related to the misconception regarding the solution to Schrödinger's equation mentioned above. While, in the context of vibrational spectroscopy, the energy eigenvalues of the individual states can be directly linked to the wave number of the absorbed photons, analysis of the wave functions (particularly the transition dipole moments and polarizabilities) is required to calculate infrared intensities and Raman activities.

Thus, avoiding a rigorous quantum mechanical treatment by sacrificing proper orthogonality may result in an accelerated framework to access wave numbers, but any calculation of vibrational transitions will ultimately appear unfeasible or – in the best case – be highly inaccurate. Although a very robust framework was established decades ago for the prediction of fundamental transitions (*i.e.* from the ground state to the first excited state), most modern research into the calculation of vibrational properties focuses on higher-order transitions relevant for IR and Raman spectroscopy in near-infrared regime. The latter are fundamentally more complex, but also provide significantly more data on chemical systems, as overtone and combination bands are accessible. A theoretical treatment of this highly complex phenomena requires access to the associated intensities / activities.

From the organisation of the manuscript, it seems that the authors are unaware of this issue, or are ignoring its implications. Due to the proposed methodology's inability to provide properly orthogonalised wave functions, which is inherent in the design of the neural network, the article must be considered as a proof of concept for a rather questionable methodology. A number of more suitable approaches are currently emerging in the contributions by different research groups (e.g. Refs 15 to 26 in this work).

2) Inadequate Example Systems: In the article, the authors discuss results for individual states of two simple molecules being CH_4 and C_3H_8. By contrast, the vast majority of authors in this field compare their results to reference data of well-established benchmark sets, primarily the VIBFREQ1295 [1] and V30 [2] benchmarks, or the work of Barone *et al.* [3]. The authors should have considered calculating data for a few representative examples, or at least comparing their data to the published values for CH_4.

As such, the presentation of individual excitation appears arbitrary. Similarly, comparison to experiment is in many cases misleading (*“correct result for wrong reason“*) and the authors are highly encouraged to adhere to the “*best practice*“ standards of the vibrational spectroscopy community.

3) Ignoring/Misciting literature: On page 2, the authors provide a bulk citation of previous works:

> *“In the past decades, neural networks have also been used to solve vibrational SE, however, mostly for synthetic systems such as one or multi-dimensional harmonic oscillators (15-26).“*

This statement is imprecise and does not do justice to the contributions made to these works. For instance:

– Lagaris *et al.* (Ref. 16) included results obtained for a three-dimensional system of coupled anharmonic oscillators, which is comparable to the treatment of a non-linear three-atomic system.

– Nakanishi and Sugawara (Ref. 17) were considering a 1D double-well potential linked to quantum tunneling in addition to the harmonic oscillator.

– Mills and coworkers (Ref. 20) demonstrated their methods including 2D double-well inverted Gaussian potentials as well as entirely random 2D potential grids.

– Hong *et al.* (Ref. 23) applied their functional tensor network to a three-body coupled oscillator, thus giving rise to correlation and entanglement.

– Gamper *et al.* (Ref. 24) applied a PINN to 1D anharmonic oscillators, rigid rotors, periodic finite potentials as well as the hydrogen molecule and the torsional tunneling of the phenole molecule.

– Domingo et al. (Ref 25) considered 1D anharmonic and polynomial potentials.

Underselling the contributions of other research groups in this way is certainly not adequate and is unacceptable in any scientific article, regardless of the journal in question.

In addition to these oversights, a plethora of literature exists on accurate, grid-based methods focused on the precise calculations of Schrödinger's equation, including many contritbutions to problems associated to molecular vibrations. This fundamental research area is not mentioned at all in the manuscript.

4) Wrong use of scientific terms: I was surprised to read the term '*asymmetric stretch*' in a contribution submitted to a Nature journal. The correct term is '*antisymmetric stretch*', since these contributions arise from an antisymmetric superposition of the isolated stretch modes of the associated bonds (*i.e.* opposite symmetry). In contrast, '*asymmetric*' implies a complete absence of any kind symmetry.

The authors will certainly argue that it is acceptable to use an incorrect scientific term because it is commonly used by the majority of researchers in the field of chemistry. I sharply disagree here. When considering contributions by leading researchers in the field, such as Yukihiro Ozaki [4], Guntram Rauthut [5] and Vincenzo Barone [6], one consistently encounters the correct term '*antisymmetric*'. There is even a dedicated contribution by a former associate editor of the journal *Applied Spectroscopy* entitled '*Molecular vibrations are not asymmetric*' [7], discussing the origins of the correct term at great length. Also companies such as Bruker are committed to establishing a correct terminology, as can be seen from their online tutorial, '*Guide to Infrared Spectroscopy*' [8].

Given that the anti-symmetric superposition arises directly from degeneracy in the quantum mechanical description of oscillator wave functions associated with the isolated stretch vibrations, the use of the wrong terminology in a scientific contribution focused on a novel treatment of Schrödingers equation for vibrational problems is not without a certain irony.

Taking into account this particular shortcoming in view of the conceptual errors highlighted above, it appears that the authors are not particularly familiar with the underlying theoretical foundations and the best practice adopted by leaders in the field. As a consequence, this manuscript is not acceptable for publication in any peer-reviewed scientific journal focused on chemical theory.

5) Missing Description of the Methodology: Although the authors explained their choices regarding the neural network in great detail, the main article does not provide a number of key pieces of information. These include:

– Details on the calculation of the potential energy surface. The authors mention the use of diffusion Monte Carlo, however, the applied software, the calculation settings or detailed reference data are not provided at all.
– No discussion regarding the actual implementation is provided in the main article. This renders statements for routines such as Fit(16200, 45, 45, 5) and Train(18600, 45, 45, 5) frequently encountered in the article inappropriate. While Fit() is briefly mentioned on page 5, it is defined as Fit(N, n_1, n_2), *i.e.* using three arguments and not four. Most likely, the authors forgot to include n_0 in the defintion. In contrast to Fit(), the routine Train() is not defined at all.

Very little information is provided in the supplementary material, so a number of key settings are missing and must be discussed in the main article in order for readers to follow the methodology, which currently is not possible.

6) Minor points:

– On page 7 the authors note for the treatment of CH_4:

  "The input layer has 10 nodes, equal to the number of bond lengths in the molecule."

  Most chemists will unanimously reject this statement, since there are only 4 bonds in the methane molecule. Most likely the authors also consider the H-H vectors as chemical bonds, which is not a correct way to describe the molecule, adding to the list of oversights and shortcomings regarding chemical terminology.
– Page numbers: The submission of the article with missing page numbers is a clear indication that the authors did not take the preparation of the article too seriously. This is inline with the many shortcomings discussed above and one can get the impression, that the entire project was considered as a quick contribution to ride on the current machine learning/AI hype. The missing quality in manuscript preparation just further underlines this point.
– Level of English: While throughout large parts of the manuscript the level of English is adequate, there are still a few sentences with missing articles. Considering that we now live in the age of AI, an automated English check with any free AI agent should be a matter of minutes.
– References: Individual references (38, 41) are incomplete adding to the picture of a quickly prepared study. Also, considering the magnitude and importance of this work, a total reference count of 41 appears to small. The authors are encouraged to maximize the allowed number of references.

In summary, this article presents an ill-conceived neural network implementation enabling the calculation of vibrational eigenenergies based on the Schrödiner equation. While being applicable to larger systems, the sacrifice of wave function orthogonality will greatly limit the framework for any

useful prediction of molecular vibrational spectra in terms of intensities / activities. Added to this methodical short comings is the absence of any notable benchmarking, the use of wrong terminology, an inadequate summary of previous contributions to the field and missing details regarding the applied methodology.

From this perspective, the manuscript cannot be accepted for publication in any peer-reviewed scientific journal. Due to its many shortcomings, it cannot be improved through revision.

For these reasons, rejection is the only possible recommendation.

References:

[1] "*VIBFREQ1295: A New Database for Vibrational Frequency Calculations*" J. C. Zapata Trujillo L. K. McKemmish *J. Phys. Chem. A* 126 (**2022**) *4100 – 4122 DOI: 10.1021/acs.Jpca.2c01438*

[2] "*The V30 benchmark set for anharmonic vibrational frequencies of molecular dimers*" J. Hoja, D. A. Boese *J. Chem. Phys.* 161 (**2024**) *234110 DOI: 10.1063/5.0238491*

[3] "*Accuracy Meets Interpretability for Computational Spectroscopy by Means of Hybrid and Double-Hybrid Functionals*" V. Barone, G. Ceselin, M. Fusè, N. Tasinato *Front. Chem.* 8 (**2020**) *584203 DOI:* 10.3389/fchem.2020.584203

[4] "*Two-Dimensional Correlation Analysis, Fourier Self-Deconvolution, and Second-Derivative Studies on Assignments of the CH_2 Stretching Bands for Cast Films of 2-Dodecyl-, 2-Pentadecyl-, and 2-Octadecyl-7,7,8,8-Tetracyanoquinodimethane*" H.-S. Wang, Y. Ozaki *Appl. Spectrosc.* 54 (**2000**) *692 – 698 DOI: 10.1366/0003702001949994*

[5] "*A combined variational and perturbational study on the vibrational spectrum of P_2F_4*" G. Rauhut, T. Hrenar *Chem. Phys.* 346 (2008) *160 – 166 DOI: 10.1016/j.chemphys.2008.01.039*

[6] "*Interplay of Stereoelectronic and Vibrational Modulation Effects in Tuning the UPS Spectra of Unsaturated Hydrocarbon Cage Compounds*" L. Paoloni, M. Fusè, A. Baiardi, V. Barone J. Chem. Theory Comput. 16 (**2020**) *5218 – 5226 DOI: 10.1021/acs.jctc.0c00645*

[7] "*Molecular Vibrations Are Not Asymmetric*" J. A. de Haseth *Appl. Spectrosc.* 76 (2022) *150 – 152* DOI: https://doi.org/10.1177/000370282110365

[8] "*Guide to Infrared Spectroscopy*" *Bruker* https://www.bruker.com/en/products-and-solutions/infrared-and-raman/ft-ir-routine-spectrometer/what-is-ft-ir-spectroscopy.html; (02.08.2025)
